# Regulation of Heterochromatin Assembly on Unpaired Chromosomes during *Caenorhabditis elegans* Meiosis by Components of a Small RNA-Mediated Pathway

**DOI:** 10.1371/journal.pgen.1000624

**Published:** 2009-08-28

**Authors:** Xingyu She, Xia Xu, Alexander Fedotov, William G. Kelly, Eleanor M. Maine

**Affiliations:** 1 Department of Biology, Syracuse University, Syracuse, New York, United States of America; 2 Biology Department and Program in Genetics and Molecular Biology, Emory University, Atlanta, Georgia, United States of America; The University of North Carolina at Chapel Hill, United States of America

## Abstract

Many organisms have a mechanism for down regulating the expression of non-synapsed chromosomes and chromosomal regions during meiosis. This phenomenon is thought to function in genome defense. During early meiosis in *Caenorhabditis elegans*, unpaired chromosomes (e.g., the male X chromosome) become enriched for a modification associated with heterochromatin and transcriptional repression, dimethylation of histone H3 on lysine 9 (H3K9me2). This enrichment requires activity of the cellular RNA-directed RNA polymerase, EGO-1. Here we use genetic mutation, RNA interference, immunofluorescence microscopy, fluorescence *in situ* hybridization, and molecular cloning methods to identify and analyze three additional regulators of meiotic H3K9me2 distribution: CSR-1 (a Piwi/PAZ/Argonaute protein), EKL-1 (a Tudor domain protein), and DRH-3 (a DEAH/D-box helicase). In *csr-1*, *ekl-1*, and *drh-3* mutant males, we observed a reduction in H3K9me2 accumulation on the unpaired X chromosome and an increase in H3K9me2 accumulation on paired autosomes relative to controls. We observed a similar shift in H3K9me2 pattern in hermaphrodites that carry unpaired chromosomes. Based on several assays, we conclude that ectopic H3K9me2 accumulates on paired and synapsed chromosomes in these mutants. We propose alternative models for how a small RNA-mediated pathway may regulate H3K9me2 accumulation during meiosis. We also describe the germline phenotypes of *csr-1*, *ekl-1*, and *drh-3* mutants. Our genetic data suggest that these factors, together with EGO-1, participate in a regulatory network to promote diverse aspects of development.

## Author Summary

DNA within a cell's nucleus is packaged together with proteins into a higher order structure called chromatin. In its simplest form, chromatin consists of DNA and a set of proteins called histones, arranged so that the DNA strand is wrapped around histone protein clusters. This basic chromatin structure can be modified in various ways to regulate access to the genetic information encoded in the DNA. Such regulation is critical for cellular function and development of the organism. As cells form gametes, they undergo a specialized type of cell division called meiosis. During meiosis, chromatin is regulated in specific ways to ensure proper development of the embryo. During meiosis in the nematode *C. elegans*, the chromatin structure of the single male X chromosome depends on an RNA-directed RNA polymerase called EGO-1. Here, we identify three more regulators of meiotic chromatin, the proteins CSR-1, EKL-1, and DRH-3. Our data suggest that these proteins collaborate with EGO-1 to ensure that paired chromosomes (autosomes and hermaphrodite X chromosomes) are regulated correctly and in a manner distinct from the male X chromosome. Our findings suggest that these four proteins participate in a mechanism to ensure proper gene expression for gamete formation.

## Introduction

During sexual reproduction, mutations existing in the gametes will be inherited by the offspring. Therefore, it is essential that gametes contain accurate copies of the genetic information. Through evolution, multiple mechanisms have been developed to safeguard gamete quality. One such mechanism may be a process referred to as meiotic silencing of unpaired chromatin (MSUC) whereby genes located on unpaired chromatin are silenced during first meiotic prophase. This is a widespread phenomenon that has been described in fungi, nematodes, and mammals (for reviews, see [1]–[3]). While it naturally involves sex chromosomes in the heterogametic sex, meiotic silencing also targets unsynapsed regions that may be present due to mutation or chromosome rearrangement [4]–[6]. MSUC may function as a surveillance mechanism to protect against detrimental conditions such as aneuploidy or expression of genetic parasites (e.g., transposable elements) that are inserted in one homolog and would not properly align during meiosis [1],[7]. MSUC may also function in the segregation of non-homologous chromosomes, e.g., the mammalian X and Y chromosome [3].

Distinct mechanisms of MSUC appear to function in different species, although some common components and features are involved. MSUC in nematodes and mammals occurs at the transcriptional level. In *C. elegans*, regions of unpaired chromatin, e.g. the male X chromosome, accumulate a histone modification associated with transcriptional silencing, H3K9me2 [4]. High levels of H3K9me2 also accumulate on free chromosomal duplications and chromosomes that fail to synapse due to mutations in both the XX and XO germ line [4]. Few X-linked genes are expressed in the male germ line, therefore it is difficult to correlate H3K9me2 accumulation with repression of gene expression in males. However, transcription of X-linked oogenesis-specific genes decreases dramatically in sexually transformed XO hermaphrodites, suggesting that the H3K9me2 marks indeed correlate with gene silencing [8]. The *C. elegans* meiotic silencing machinery may involve small RNA, e.g., small interfering (si) RNA, as activity of the RNA-directed RNA polymerase (RdRP), EGO-1, is required for H3K9me2 enrichment on unpaired regions [9]. In mouse, as in *C. elegans*, histone modifications associated with gene silencing accumulate on regions of unpaired chromatin, e.g. the male X and Y chromosomes and chromosomal translocations in both XX and XO germ lines. These regions also accumulate histone variants, e.g., macroH2A1.2 and γH2AX [5], [10]–[13,] (see also [3]). Mammalian meiotic silencing is known to require machinery closely related to the DNA repair pathways (see [1]). In the filamentus fungus, *Neurospora crassa*, MSUC (also called meiotic silencing by unpaired DNA, MSUD) requires the activity of: an RNA-directed RNA polymerase (RdRP), SAD-1 [14]; a member of the Argonaute family of RNA binding proteins, SMS-2 [15]; and a Dicer endonuclease-like protein, DCL-1 [16]. *N. crassa* meiotic silencing appears to occur at a post-transcriptional level via a mechanism related to RNA interference (RNAi) (for a review of the core RNAi machinery, see [17]). For example, meiotic silencing elicited by a chromosomal deletion will target paired copies of the deleted region (present as transgenes) that are located at a distinct site [14]. This behavior is not observed in *C. elegans*, e.g., the presence of a chromosomal duplication does not lead to H3K9me2 accumulation on paired copies of the intact chromosome [4],[8].

Small RNA has been implicated in heterochromatin assembly in a number of systems (for reviews, see [18]–[20]). Mechanistic details appear to vary from one system to another, as the mechanisms involve different constellations of proteins. The best-studied case, heterochromatin assembly at centromeric repeats in the yeast, *Schizosaccharomyces pombe*, requires Dicer, RdRP, and Argonaute (Ago) activity [21]–[25]. Here, Ago and endogenous siRNAs participate in an RNA-induced transcriptional silencing (RITS) complex whose chromatin association is sufficient for directing H3K9me2 accumulation [18],[19],[25]. Dicer and Argonaute activity have also been shown to promote centromeric heterochromatin assembly in *Drosophila melanogaster*, an organism that apparently lacks cellular RdRP [26],[27]. Similarly, Dicer, Argonaute, and RNA helicase activities are linked to heterochromatin formation and the subsequent elimination of repeated DNA sequences in the micronuclei of *Tetrahymena thermophila* [28],[29] (for a review, see [30]). In mammals, as well, a growing body of evidence suggests that promoter transcripts and an Argonaute protein may participate in transcriptional regulation [31]–[33]. In general, these transcriptional silencing mechanism(s) are poorly understood, and the identified RNAi factors might act indirectly, e.g., as participants in the post-transcriptional regulation of genes whose products function directly in chromatin regulation. Interestingly, Dicer activity does not appear to be required for meiotic H3K9me2 enrichment on unpaired chromatin in *C. elegans*, suggesting that microRNAs and other Dicer-dependent RNA products do not participate in the regulatory process [9].

To identify additional components of the MSUC machinery in *C. elegans*, we surveyed candidate genes to identify those whose loss of function altered the pattern of H3K9me2 accumulation during meiosis. Here, we report the identification of CSR-1, EKL-1, and DRH-3 as additional regulators of meiotic H3K9me2 accumulation. These proteins function in RNAi, and DRH-3 (like EGO-1) is implicated in the biogenesis of endogenous siRNAs [34],[35]. Here, we provide evidence that H3K9me2 does not accumulate properly on unpaired chromatin in *csr-1*, *ekl-1*, and *drh-3* mutants and is mis-targeted to correctly paired and synapsed chromatin. Moreover, the germline phenotypes of *csr-1*, *ekl-1*, and *drh-3* mutants are complex and share some features with the *ego-1* phenotype. As previously shown for *ego-1* [36],[37], *csr-1*, *ekl-1*, and *drh-3* interact genetically with *glp-1*, which encodes the germline Notch-type receptor required for germ cell proliferation. We discuss alternative models for how these factors may participate in the regulation of meiotic chromatin.

## Results

### Identification of new regulators of meiotic H3K9me2 distribution

We used two approaches to identify candidate genes whose products might participate in meiotic silencing: (i) we compiled a list of factors that had been implicated in small RNA-mediated processes, including Argonaute proteins and putative chromatin-associated proteins [38]–[52] (Table 1); and (ii) we surveyed a set of *ego* mutants, previously isolated in our screens for genetic **e**nhancers of ***g*** *lp-* ***1*** (*ego* mutations), whose phenotypes resemble that of *ego-1* [36] (J. Spoerke and E. Maine, unpublished data). *ego-1* mutants have a specific developmental phenotype that is not commonly observed, but is characteristic of some other mutants isolated in our *ego* screens.

**Table 1:** Candidate genes surveyed for H3K9me2 and Ego phenotypes.

Annotation	Gene	Protein family/domain	Small RNA processes [ref]	Δ H3K9me2	Ego
C01G5.2	*prg-2*	Piwi-AGO Family	Tc3 silence [41]	−	−
C04F12.1		Piwi-AGO Family	Soma RNAi [39],[42]	−	−
C16C10.3		Piwi-AGO Family	NR	−	−
C18E3.7	*ppw-1*	Piwi-AGO Family	Gln RNAi, T silence [42],[43]	−	−
D2030.6	*prg-1*	Piwi-AGO Family	Tc3 silence, 21U-RNA [41], [43]–[45]	−	−
F20D12.1	*csr-1*	Piwi-AGO Family	Co-sup, RNAi, Slicer [40],[42],[46]	+	+
F55A12.1		Piwi-AGO Family	NR	−	−
F56A6.1	*sago-2*	Piwi-AGO Family	Soma RNAi [42]	−	−
F58G1.1		Piwi-AGO Family	Gln/soma RNAi [42]	−	−
K12B6.1	*sago-1*	Piwi-AGO Family	Soma RNAi [40],[42]	−	−
R04A9.2	*nrde-3*	Piwi-AGO Family	siRNA nuclear import [47]	−	−
R06C7.1		Piwi-AGO Family	NR	−	−
R09A1.1	*ergo-1*	Piwi-AGO Family	endo-RNAi [42]	−	−
T07D3.7	*alg-2*	Piwi-AGO Family	miRNA function [48]	−	−
T22B3.2		Piwi-AGO Family	NR	−	−
T22H9.3		Piwi-AGO Family	NR	−	−
T23D8.7		Piwi-AGO Family	NR	−	−
Y110A7A.18	*ppw-2*	Piwi-AGO Family	Co-sup, T silence [40],[49]	−	−
Y49F6A.1		Piwi-AGO Family	NR	−	−
ZK1248.7		Piwi-AGO Family	NR	−	−
ZK218.8		Piwi-AGO Family	NR		
ZK757.3		Piwi-AGO Family	NR	−	−
C01B10.1	*drh-2*	DExH-box helicase	RNAi [50]	−	−
D2005.5	*drh-3*	DEAH/D-box helicase	RNAi, siRNA biogenesis [34],[46]	+	+
F15B10.2	*drh-1*	DExH-box helicase	RNAi [39],[50]	−	−
C08B11.2	*hda-2*	Class I HDAC	NR	−	ND
C10E2.3	*hda-4*	Class II HDAC	NR	−	ND
C35A5.9		Class IV HDAC	NR	−	ND
D2096.8		NAP-related	RNAi, T silence [39],[49]	−	−
F02E9.4	*sin-3*	SIN3 HDAC	RNAi [39]	+	−
F22D6.6	*ekl-1*	Tudor domains	Co-sup, RNAi [39],[40]	+	+
F45E4.9	*hmg-5*	HMG box	Co-sup [36]	−	−
F54F2.2	*zfp-1*	Various^1^	RNAi [39],[51]	−	−
K04G7.3	*ogt-1*	See footnote^2^	Predicted SIN-3 interactor [52]	−	−
M04B2.3	*gfl-1*	See footnote^3^	RNAi [39],[51]	−	−
T22B7.1	*egl-13*	HMG-box^4^	Co-sup [40]	−	−
W02D9.8		HMG box	Co-sup [40]	−	−
ZK1127.7	*cin-4*	DNA topo IIA^5^	RNAi [39]	−	−
T09E8.1		Novel^6^	Co-sup [40]	−	−

All genes were tested using a deletion mutant (see Materials and Methods) except for the following, for which we knocked down the protein using RNAi: C08B11.2/*hda-2*, C10E2.8/*hda-4*, C35A5.9, K04G7.3/*ogt-1*, T22B7.1/*egl-13/cog-2*, and ZK1127.7/*cin-4*. Ego phenotype means all germ cells prematurely exited mitosis, entered meiosis, and underwent gametogenesis. Δ H3K9me2 means a change in the distribution and/or level of H3K9me2 in the meiotic germ line. We previously reported that mutations in the Argonaute genes *rde-1* and *alg-1* did not visibly alter the pattern of H3K9me2 accumulation during meiosis [9]. Additional putative Piwi/Argonaute genes, previously reported in the literature and now thought to be pseudogenes, are not listed here (http://www.wormbase.org). Gln, germ line; Co-sup, co-suppression; T silence, transposon silencing; Tc3 silence, Tc3 silencing; 21U-RNA, 21U-RNA regulation; HDAC, histone deacetylase; NAP, nucleosome assembly protein. NR, none reported.

1 Contains leucine zipper, Zinc finger, and PHD/LAP domains; homologous to human AF10.

2 O-linked N-acetylglucosamine transferase.

3 Homology to human glioma-amplified sequence 41, yeast transcription factor AF-9, and human transcription factor ENL.

4 Related to transcription factor SOX5.

5 DNA topoisomerase, type IIA.

6 Loss of gene function causes sterility.

We subjected these candidates to two tests. First, we used indirect immunofluorescence to evaluate the meiotic H3K9me2 staining pattern in available mutants or after depletion via RNAi (see Materials and Methods). Our RNAi assays were performed using *him-8* mutants, as the hermaphrodite X chromosomes remain unpaired/unsynapsed and therefore become enriched for H3K9me2. Second, we tested type (i) candidates genes for an Ego phenotype using RNAi-mediated knockdown in animals with a weak *glp-1* loss of function mutation, *glp-1(bn18ts)* at 20°C (see Materials and Methods).

**Figure 1:**
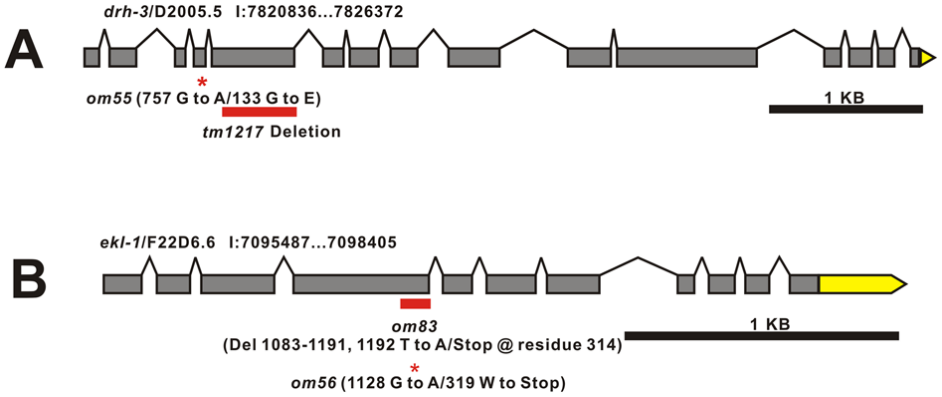
*drh-3* and *ekl-1* mutations isolated as genetic enhancers of *glp-1*. The locations of *ego* alleles within (A) *drh-3* and (B) *ekl-1* are shown. *ego* alleles were isolated in screens for genetic enhancers of *glp-1* sterility. Annotated open reading frame designation (e.g., D2005.5) and nucleotide position on LGI (e.g., I:7820836) are indicated. Boxes represent exons and lines represent introns. Nucleotide and amino acid changes are indicated. The position of the *drh-3(tm1217)* deletion is indicated for reference. 3′ UTRs are shaded yellow; 5′ UTRs are not represented.

We identified four genes from the candidate gene list whose activities influenced meiotic H3K9me2 distribution: *csr-1*, *ekl-1*, *drh-3*, and *sin-3* (Table 1). Three of these genes were also identified as Ego: *csr-1*, *ekl-1*, and *drh-3* (Table 1). We also identified three *ego* mutants with altered H3K9me2 distribution, *ego(om55)*, *ego(om56)*, and *ego(om83)*. The three *ego* mutations mapped close to *ekl-1* and *drh-3* (see Materials and Methods); we cloned them in order to determine whether they represented alleles of *ekl-1*, *drh-3*, and/or other genes whose products function in meiotic chromatin regulation. Our data indicate that *ego(om56)* and *ego(om83)* are alleles of *ekl-1*, and *ego(om55)* is an allele of *drh-3* (Figure 1) (see Materials and Methods). Intriguingly, CSR-1 (an Argonaute protein), DRH-3 (a Dicer-related DEAH/D-box helicase), and EKL-1 (a Tudor domain protein) all, like EGO-1, promote RNAi (Table 1). Hence, we hypothesize that these factors may work together to regulate meiotic H3K9me2 accumulation via a mechanism that involves small RNA, e.g., endogenous siRNA. We will focus this report on the role of CSR-1, EKL-1, and DRH-3 in meiotic silencing and will discuss SIN-3 in a future report.

### Meiotic H3K9me2 distribution in *csr-1*, *ekl-1*, and *drh-3* mutants

**Figure 2:**
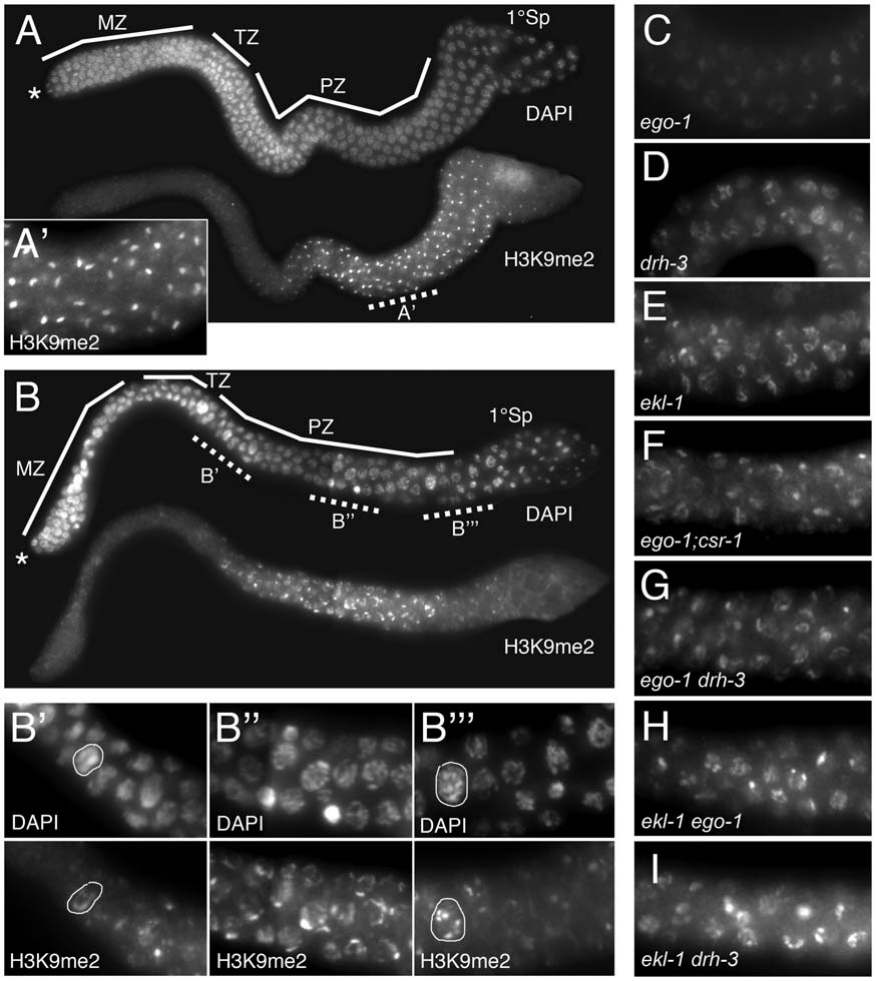
Abnormal H3K9me2 accumulation in *csr-1*, *ekl-1*, and *drh-3* mutants. Each panel shows germline nuclei co-labeled with DAPI to visualize DNA and polyclonal anti-H3K9me2 antibody. Tissue was dissected and fixed at 24 hr post-L4 stage. The distal-to-proximal axis is oriented left to right in each image. All images were taken at the same exposure. (A and B) Features of the wild type (N2) and *csr-1(tm892)* male germ lines are shown. Distal ends (asterisk), mitotic zones (MZ), transition zones (TZ), pachytene zones (PZ), and primary spermatocytes are indicated. In both germ lines, H3K9me2 is first detected at early pachytene stage. (A') In the N2 germ line, a single strong focus of H3K9me2 labeling corresponds to the single X chromosome (as described in [4]); other chromosomes accumulate a low level of H3K9me2 (as described in [9]). (B) In the *csr-1(tm892)* germ line, H3K9me2 labeling is more broadly distributed than in wildtype; a single strong focus of labeling is often absent. (B') An example of a large, morphologically abnormal nucleus is circled. See Results. (B'″) As in wildtype, H3K9me2 levels decrease as germ cells become primary spermatocytes. Circled nucleus has the “elevated” H3K9me2 pattern referred to in Table 2 and described in Results. (C–I) Each panel shows H3K9me2 distribution in pachytene nuclei at a position corresponding to the region shown in (B″-B'″). (C) The X chromosome fails to accumulate a high level of H3K9me2 in *ego-1(om84)* meiotic nuclei; a basal level of H3K9me2 is broadly distributed over all chromosomes. (D,E) H3K9me2 distribution in *drh-3(tm1217)* and *ekl-1(om56)* single mutants resembles that observed in *csr-1(tm892)* animals (see (B″)). (F–I) H3K9me2 distribution in *ego-1;csr-1*, *ego-1 drh-3*, *ekl-1 ego-1*, and *ekl-1 drh-3* double mutants resembles that observed in *csr-1*, *ekl-1*, and *drh-3* single mutants. Images were captured on a Zeiss Axioscope.

**Figure 3:**
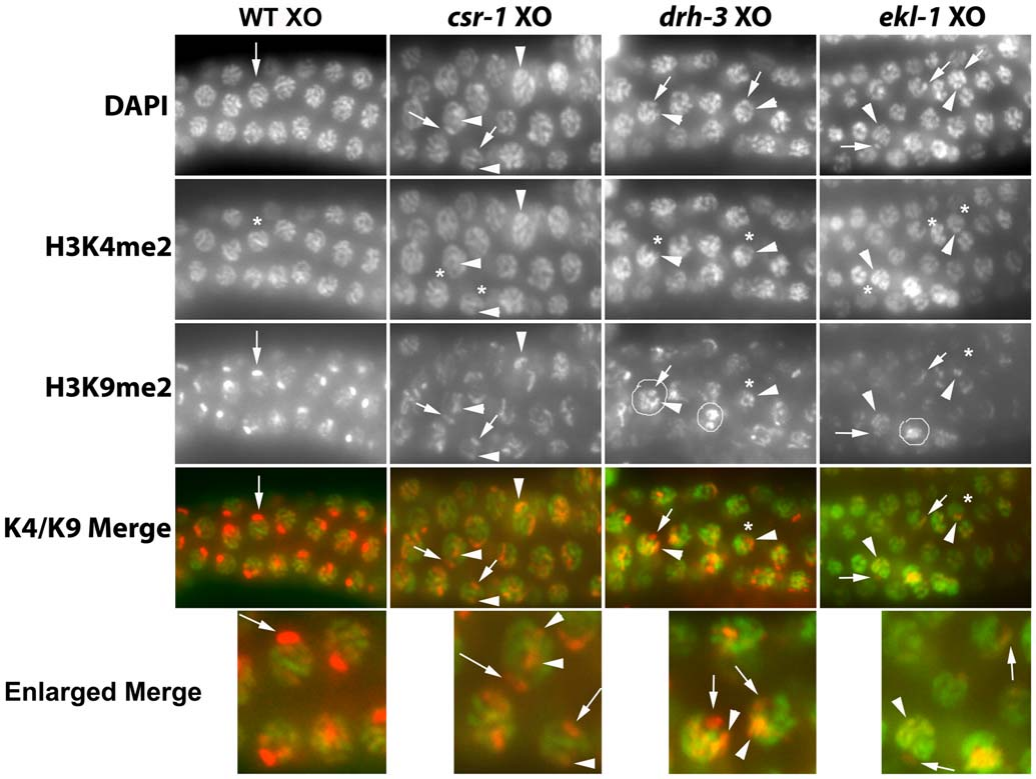
Relative distribution of H3K4me2 and H3K9me2 marks during XO meiosis. Each panel shows meiotic nuclei co-labeled with DAPI to visualize DNA, polyclonal antibody against H3K4me2, and monoclonal antibody against H3K9me2. In wildtype germ cells, the X chromosome (arrow) lacks H3K4me2 (*) and becomes highly enriched for H3K9me2 (arrow). In contrast, autosomes become highly enriched for H3K4me2 and accumulate a very low level of H3K9me2. In *csr-1*, *ekl-1*, and *drh-3* nuclei, one chromosomal region lacks H3K4me2 (*) and contains a variable level of H3K9me2 (arrow); this is presumably the X chromosome. In some cases, the X chromosome lacks detectable H3K9me2 altogether (*). Other chromosomes contain substantial H3K4me2 and a variable level of H3K9me2 (arrowheads). Circled nuclei are examples of the “elevated” H3K9me2 pattern referred to in Table 2; most other nuclei have the “dispersed” H3K9me2 pattern referred to in Table 2. See Results. Images were captured on a Zeiss Axioscope at the same exposure and processed in a similar manner.

In wild type males, the X chromosome preferentially becomes enriched for H3K9me2 during early pachytene stage and maintains this enrichment until germ cells become primary spermatocytes [4] (Figure 2A). Other chromosomes exhibit a relatively low level of H3K9me2. In *ego-1* null mutant males (hereafter designated *ego-1* males), X chromosome enrichment fails to occur, and all chromosomes accumulate a variable and low level of H3K9me2 [9] (Figure 2C). In *csr-1(tm892)*, *ekl-1(om83)*, and *drh-3(tm1217)* males, H3K9me2 was distributed more broadly across the chromosomes than in controls, and a single focus was rarely observed (Figure 2B, 2D, 2E). Several foci were visible in some nuclei, which also tended to have a higher overall level of the mark. We quantified the relative proportion of nuclei with each labeling pattern, and the proportion of nuclei with normal meiotic *versus* abnormal chromosomal morphology (Table 2). It was difficult to quantify the H3K9me2 level since the labeling intensity varied even among control preparations. However, we obtained a general measure of labeling intensity by comparing images captured at equivalent exposures. The majority of nuclei with normal pachytene morphology lacked a strong focus of H3K9me2 labeling when compared with wild type (ranging from 57% of pachytene nuclei in *csr-1* males to 83% of pachytene nuclei in *ekl-1* males) (Figure 3). A smaller proportion of the nuclei with normal morphology had multiple bright H3K9me2 foci and/or a higher overall level of H3K9me2 (ranging from 14% in *ekl-1* to 34% in *csr-1* males) (Figure 2B, Figure 3). In such nuclei, one of the foci may correspond to the X chromosome. The H3K9me2 distribution appeared essentially normal in a small proportion of nuclei (ranging from 3% in *ekl-1* to 9% in *csr-1* males), particularly nuclei located in the proximal region of the gonad arm. Among morphologically abnormal nuclei, the most striking ones were large and had diffuse chromosome morphology; these nuclei, which may have been polyploid, tended to have multiple H3K9me2 foci and a high overall level of H3K9me2 (Figure 2B, Table 2).

**Table 2:** Meiotic H3K9me2 distribution patterns in *csr-1*, *ekl-1*, and *drh-3* mutant males.

Genotype	Nuclear morphology	H3K9me2 label:	Elevated	Dispersed	1 focus	N
***csr-1***	pachytene^1^		34%	57%	9%	164
	abnormal^2^		78%	22%	0%	18
***ekl-1;him-8***	pachytene^1^		14%	83%	3%	216
	abnormal^2^		85%	15%	0%	13
***drh-3;him-8***	pachytene^1^		23%	65%	11%	126
	abnormal^2^		80%	20%	0%	5

See Results and Figures 2 and 3 for descriptions and representative images of the three H3K9me2 distribution patterns (elevated, dispersed, and single focus). “1 focus” indicates that a single strong focus of H3K9me2 labeling was observed. N, number of nuclei counted.

1 These nuclei have recognizable pachytene morphology.

2 These abnormal nuclei are large and have a diffuse chromosomal morphology not typical of meiosis. The percent of nuclei within the pachytene zone with this morphology was: 10% for *csr-1*; 6% for *ekl-1;him-8*; and 4% for *drh-3;him-8*.

To identify the X chromosome, we co-labeled H3K9me2 and a histone “activating” mark that is present on autosomes but absent from the X chromosomes in germ cells, H3K4me2 [4],[53]. We consistently observed a chromosome without H3K4me2, which presumably corresponds to the X chromosome (Figure 3). We observed a variable level of H3K9me2 associated with this chromosome; in many nuclei, the level was substantially reduced compared with controls. We also observed frequent H3K9me2 enrichment co-localizing with H3K4me2 (Figure 3). We interpreted this phenotype to reflect enrichment for H3K9me2 on autosomal sites in the absence of CSR-1, EKL-1, or DRH-3 activity.

**Figure 4:**
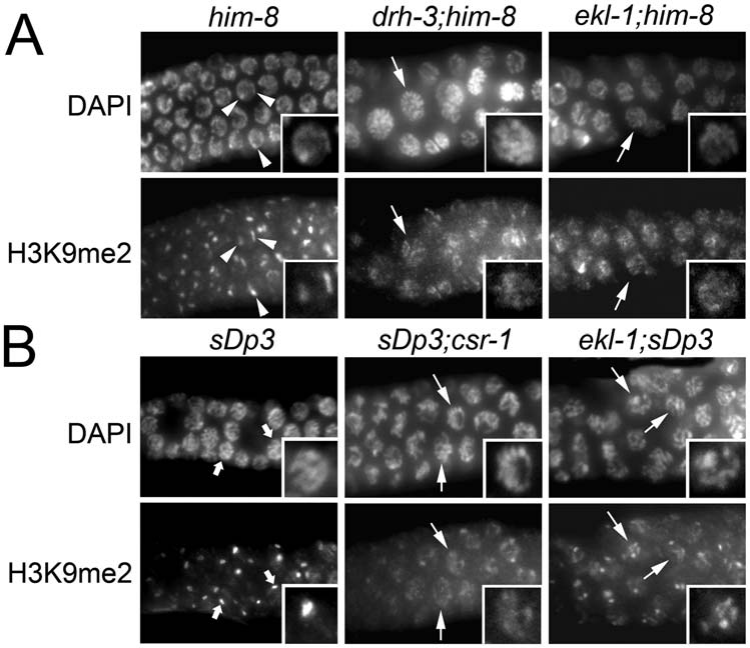
Reduced H3K9me2 levels in *csr-1*, *ekl-1*, and *drh-3* hermaphrodites carrying unpaired X chromosomes or a chromosomal duplication. Each panel shows hermaphrodite germline nuclei co-labeled with DAPI to visualize DNA and with polyclonal anti-H3K9me2 antibody. All images were taken at the same exposure. Unpaired chromatin was introduced into hermaphrodite germ cells using (A) a *him-8* mutation to prevent X chromosome pairing or (B) the free duplication, *sDp3*. (A) In *him-8* controls, H3K9me2 foci are visible (arrowheads). In *drh-3;him-8* and *ekl-1;him-8* mutants, unpaired X chromosomes fail to become enriched for H3K9me2 (arrows). (B) In control nuclei, H3K9me2 is elevated on *sDp3* (fat arrows); occasional nuclei have two foci that are interpreted to reflect partial pairing of *sDp3* with an intact LGIII, resulting in H3K9me2 enrichment on the unpaired portions of *sDp3* and LGIII. In *sDp3;csr-1* and *ekl-1;sDp3* nuclei, the H3K9me2 labeling is no longer concentrated as a single strong focus, but instead is found on multiple chromosomes (arrows) and in some cases is reduced on *sDp3* relative to wild type. Images were captured on a Zeiss Axioscope.

In contrast to the male (XO) germ line, we observed no obvious defect in H3K9me2 accumulation in mutant hermaphrodite (XX) germ lines (data not shown). We considered two possibilities for why this might be the case: CSR-1, EKL-1, and DRH-3 function might affect chromatin assembly only in male germ cells or, alternatively, only in germ cells with significant unpaired chromatin (e.g., unpaired chromosomes or a chromosomal duplication). To distinguish between these hypotheses, we examined H3K9me2 accumulation in mutant hermaphrodites where the X chromosomes did not pair or synapse (genotype *ekl-1(om83); him-8* and *drh-3(tm1276); him-8*) and in hermaphrodites carrying a free chromosomal duplication (genotype *sDp3;csr-1(tm892)*, *ekl-1;sDp3*, and *drh-3;sDp3*). In these five strains, H3K9me2 foci were reduced in intensity relative to controls, and there appeared to be a mild increase in H3K9me2 levels on other chromatin (Figure 4). To distinguish between autosomes and X chromosomes in *drh-3;him-8* and *ekl-1;him-8* hermaphrodites, we co-labeled H3K4me2 and H3K9me2 marks. In both control and experimental animals, we consistently identified chromosomal regions that failed to accumulate H3K4me2 and were presumably the X chromosomes (Figure S1). Consistent with the data presented in Figure 4, these H3K4me2-negative regions were highly enriched for H3K9me2 in *him-8* controls, but much less so in the mutants. These results suggest that unpaired regions are not as highly targeted for H3K9me2 in *csr-1*, *ekl-1*, and *drh-3* hermaphrodites as they are in wildtype hermaphrodites.

### *csr-1*, *ekl-1*, and *drh-3* mutants have multiple germline developmental defects

**Figure 5:**
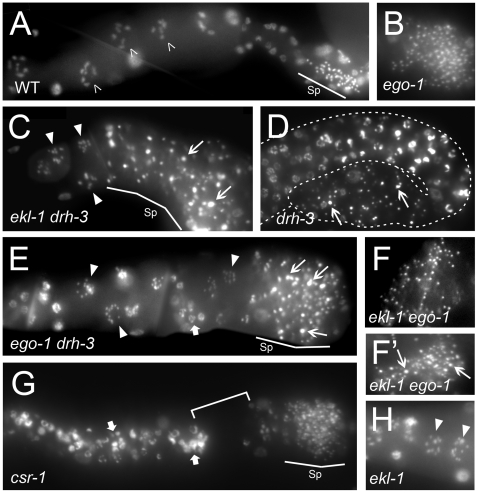
Examples of gametogenesis defects associated with loss of *csr-1*, *drh-3*, and *ekl-1* function. Each panel shows a portion of a dissected gonad arm stained with DAPI to visualize DNA. Known or putative null alleles are used in all cases. (A) Wildtype (N2) hermaphrodite with oocyte nuclei (open arrowheads) and sperm (Sp, line) are indicated. Note that sperm are essentially uniform in size. Oocyte nuclei at diakinesis have six bivalent chromosomes. (B) *ego-1* sperm are essentially uniform in size. (C) *ekl-1drh-3*, (E) *ego-1drh-3*, and (H) *ekl-1* germ lines contain small, crowded oocytes with univalent chromosomes (closed arrowheads). (C) *ekl-1drh-3*, (D) *drh-3*, (E) *ego-1drh-3*, and (F') (some) *ekl-1 ego-1* germ lines contain variably sized sperm (arrows). (F) Some *ekl-1 ego-1* germ lines contain sperm of essentially uniform size. (E and G) Morphologically abnormal nuclei are visible amidst diakinesis (oocyte) nuclei in *ego-1drh-3* and *csr-1* germ lines (fat arrows). In addition, *csr-1* meiotic nuclei are often clumped together, leaving gaps without nuclei (bracket).

We compared the developmental phenotypes of *ego-1*, *csr-1*, *ekl-1* and *drh-3* mutants in order to address further the functional relationship among the four gene products (see Materials and Methods). As discussed above, loss-of-function mutations in each gene enhanced a mild GLP-1/Notch defect in the germ line. We observed additional germline defects in young adult hermaphrodites and males of each genotype, as follows: a moderately reduced number of germ cells; a larger than normal proportion of leptotene-zygotene nuclei; a smaller than normal proportion of pachytene nuclei; a delay in the sperm-oocyte switch; and abnormal oogenesis (Figure 5D, 5G, 5H, Table 3, data not shown). *ekl-1*, *csr-1*, and *drh-3* germ lines contained some large nuclei with a diffuse chromosomal morphology quite distinct from pachytene and diplotene nuclei; we previously observed morphologically similar nuclei in *ego-1* mutants [35],[54] (see Figures 2 and 3, Table 2). 100% of hermaphrodites produced abnormal, small oocytes and 100% of their progeny died as embryos. As adults aged, oocytes tended to back up around the loop and there was a reduction in the proportion of the germ line in mitosis and first meiotic prophase. These observations are consistent with previous reports of sterility in *csr-1* mutants [40],[42], and *ekl-1* mutants [40] and an oogenesis defect in *drh-3* mutants [34],[56].

**Table 3:** Gametogenesis defects associated with *csr-1*, *ekl-1*, *drh-3*, and *ego-1* single and double mutants.

Genotype	% No oocytes (N)^1^	% Irregular sperm (N)^2^	% Univalents (N)^3^	^#^Chromosomes^4^
**Wildtype**	0 (>100)	0 (>100)	0 (>100)	NA
***ego-1***	9 (33)	0 (33)	18 (11)	7.7±0.2 (7–9)
***csr-1***	68 (34)	0 (34)	18 (11)	7.3±0.3 (7–8)
***drh-3***	44 (43)	100 (43)	9 (11)	8.4±0.4 (7–11)
***ekl-1***	24 (50)	100 (50)	50 (32)	9.7±0.2 (9–10)
***ego-1 drh-3***	40 (56)	100 (56)	100 (33)	8.6±0.3 (7–12)
***ekl-1 ego-1***	39 (36)	100 (36)*	69 (13)	9.6±0.3 (7–11)
***ego-1; csr-1***	56 (34)	6 (34)*	17 (18)	8.8±0.5 (7–12)
***ekl-1 drh-3***	27 (59)	100 (59)	92 (26)	9.0±0.4 (7–11)
***ekl-1; csr-1***	7 (42)	100 (21)	ND	ND

Alleles used were *ego-1(om84)*, *ekl-1(om83)*, *drh-3(tm1276)*, and *csr-1(tm892)*. Animals were grown at 20°C and characterized at 24 hr post-L4 stage. At a slightly later time point (66–72 hr post-L1 stage), most animals contain oocytes, hence the absence of oocytes indicates a delay in the sperm-to-oocyte switch. (N), number of germ lines counted.

1 Percent of germ lines where oocytes were absent at the time of assay; note that wildtype germ lines all contain oocytes at this stage.

2 Percent of germ lines where sperm nuclear morphology was highly irregular.

*: Abnormal sperm morphology was less severe than for the other genotypes (see Results).

3 Percent of oogenic germ lines with univalents; typically, a subset of diakinesis nuclei contained univalents and a subset did not.

4 Number of chromosomes at diakinesis in nuclei with at least one set of univalent chromosomes. Nuclei with 6 bivalents are not included in the calculation. Standard error of the mean (±^#^) is indicated. The range of observed values is indicated in brackets; e.g., among *ego-1* nuclei with univalent chromosomes, a range of 7–9 chromosomes were observed. NA, not applicable. ND, univalents were observed, but not counted.

Wild type oocytes arrest at diakinesis with six pairs of bivalents visible per nucleus (Figure 5A). In *csr-1*, *ekl-1* and *drh-3* mutants, a subset of oocyte nuclei appeared to contain unpaired homologous chromosomes (univalents), as previously observed for *ego-1* (Figure 5H, and data not shown) [37]. The penetrance of this phenotype was variable with respect to the number of univalents per nucleus and the proportion of diakinesis nuclei with this abnormal morphology. The phenotype was more penetrant in *ekl-1* mutants (50% of gonad arms contained at least one oocyte with univalents) than in *ego-1*, *csr-1*, or *drh-3* mutants (≤18% of gonad arms contained oocytes with univalents) (Table 3) [37]. The presence of univalents was rarely fully penetrant within any single oocyte; instead, for individual mutants, the number of abnormal chromosome figures ranged from 7 (*ego-1*, *csr-1*, *drh-3*) to 11 (*drh-3*) (Table 3) indicating asynapsis or desynapsis of 1–5 chromosome pairs. The presence of both a protracted leptotene-zygotene region and univalent chromosomes at diakinesis, could indicate pairing, synapsis, and/or recombination defects in these mutants [57].

Spermatogenesis in 100% of *ekl-1* and *drh-3* mutants (males and hermaphrodites) was visibly abnormal in a manner that we did not observe in *ego-1* or *csr-1* mutants (Figure 5D and 5I *versus* Figure 5B and 5G, Table 3). *ekl-1* and *drh-3* sperm nuclei were abnormally large and variably sized, as if chromatin condensation or chromosome segregation was impaired. In addition, male sperm did not become tightly packed in the vas deferens (as they do in wild type).

Analysis of double mutant phenotypes suggested a complex relationship among *ego-1*, *csr-1*, *ekl-1*, and *drh-3* with respect to germline development. (See Materials and Methods for generation of double mutants.) Several aspects of the phenotype were more severe in at least a subset of double mutants. For example, the frequency of animals with univalents at diakinesis was higher among *ekl-1 drh-3*, *ego-1 drh-3*, and *ekl-1 ego-1* double mutants than in *ekl-1*, *ego-1*, and *drh-3* single mutants reflecting either a synergistic or additive effect (Table 3). Interestingly, although the frequency of animals showing the phenotype increased, the degree of asynapsis in individual nuclei was not significantly higher in double mutants compared with single mutants (Table 3). In contrast, the univalent frequency in *ego-1;csr-1* double mutants was similar to that observed in *csr-1* and *ego-1* single mutants (Table 3). We observed the sperm condensation defect in *ekl-1 ego-1* and *ego-1 drh-3* double mutants, indicating it is epistatic to the more normal sperm morphology present in *ego-1* single mutants (Figure 5E and 5F'). Interestingly, we observed a similar, although less severe, condensation defect in a subset of *ego-1;csr-1* double mutants (Table 3). The implications of these double mutant phenotypes are considered in the Discussion.

*In situ* hybridization data compiled by the Nematode Expression Pattern Database (NEXTDB, http://nematode.lab.nig.ac.jp) are consistent with our phenotypic observations. The highest concentrations of *csr-1*, *ekl-1*, and *drh-3* transcripts were detected in the gonad and in early embryos, suggesting major functions in the germ line and early embryo. Similarly, *ego-1* mRNA is highly enriched in the germ line [37] and the NEXTDB observed *ego-1* transcripts in the gonad and early embryo. The severity of the oogenesis defect in these mutants precludes our analysis of embryonic phenotypes. However, RNAi-based surveys of gene function have reported embryonic defects associated with weak knockdown of all four genes [55]–[56], [58]–[63].

### Pairing, synapsis, and meiotic H3K9me2 levels

The presence of univalent chromosomes in *csr-1*, *ekl-1*, and *drh-3* diakinesis nuclei was particularly relevant to the H3K9me2 defect. In *C. elegans*, univalents can result from defective homolog pairing, synapsis, and/or double-strand break (DSB) formation [64],[65]. Both pairing and synapsis have been implicated as important in the process by which meiotic silencing is triggered, whereas DSB formation/repair has not: H3K9me2 enrichment is observed on autosomes in XO mutants with pairing and/or synapsis defects, but not in mutants defective only in double-strand break formation (A. Fedotov and W. Kelly, manuscript in preparation). Therefore, we considered that autosomal H3K9me2 levels might be elevated in *csr-1*, *ekl-1*, and *drh-3* mutants due to (i) mis-targeting of the chromatin-modifying machinery to inappropriate sites or (ii) appropriate targeting to autosomal regions due to a meiotic pairing and/or synapsis defect. Consequently, we decided to evaluate pairing and synapsis in these mutants. We evaluated homolog pairing using fluorescent *in situ* hybridization (FISH) to visualize the 5S ribosomal RNA gene cluster located on LGV (see Materials and Methods). We detected a minor pairing defect in *drh-3* and *ekl-1* mutants (Text S1, Table S1, Table S2, Table S3). However, the frequency of nuclei where chromosome V was unpaired was much lower than the frequency of nuclei with ectopic H3K9me2 (Text S1, Table S1, Table S2, Table S3). We concluded that H3K9me2 must have accumulated on paired chromosomes in these mutants. We investigated synaptonemal complex integrity by co-labeling two proteins involved in synapsis, HIM-3 and SYP-1 [66]–[68] (see Materials and Methods). Our data did not reveal a defect in synapsis in mutant males (Figure S2) or hermaphrodites (Figure S3, Figure S4). See Text S1 for further details.

### H3K9me2 accumulates at paired and synapsed regions in *csr-1*, *ekl-1*, and *drh-3* mutants

**Figure 6:**
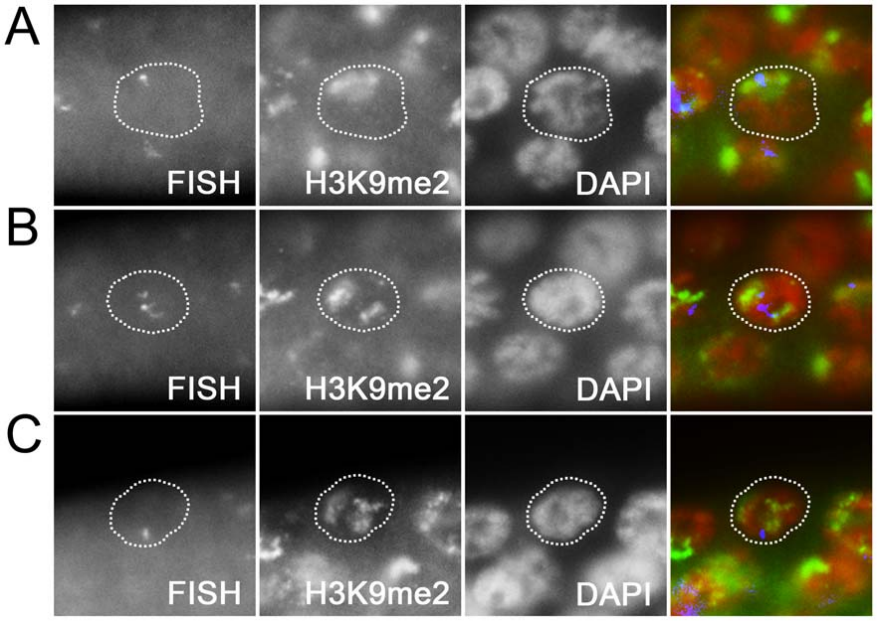
Distribution of H3K9me2 relative to 5S rDNA FISH signal. Panels show representative pachytene nuclei from a *drh-3* XO germ line co-labeled to detect 5S rDNA (by FISH) and H3K9me2. (A,C) The single FISH signal is adjacent to a region with high H3K9me2 label. (B) Two FISH signals are detected, one of which is well-separated from regions of high H3K9me2 label. Images were captured on a Leica DRMXA microscope.

To better address the relationship between pairing and H3K9me2 accumulation, we performed simultaneous LGV FISH and H3K9me2 immunolabeling on *drh-3* males. *drh-3* was chosen because it has the strongest pairing defect of the three mutants examined (Table S1, Table S2). We observed nuclei where elevated H3K9me2 and a single LGV FISH signal coincided, consistent with elevated H3K9me2 on the paired LGVs (Figure 6A, 6C). We also observed nuclei where H3K9me2 accumulated at sites distinct from one or both of two FISH signals, consistent with low H3K9me2 on unpaired chromosome Vs (Figure 6B). Given these results and the data presented in Table 2, Table S1, and Text S1, we conclude that the H3K9me2 distribution in *drh-3*, *ekl-1*, and *csr-1* pachytene nuclei is likely to be independent of the (mild) pairing defect in these mutants.

**Figure 7:**
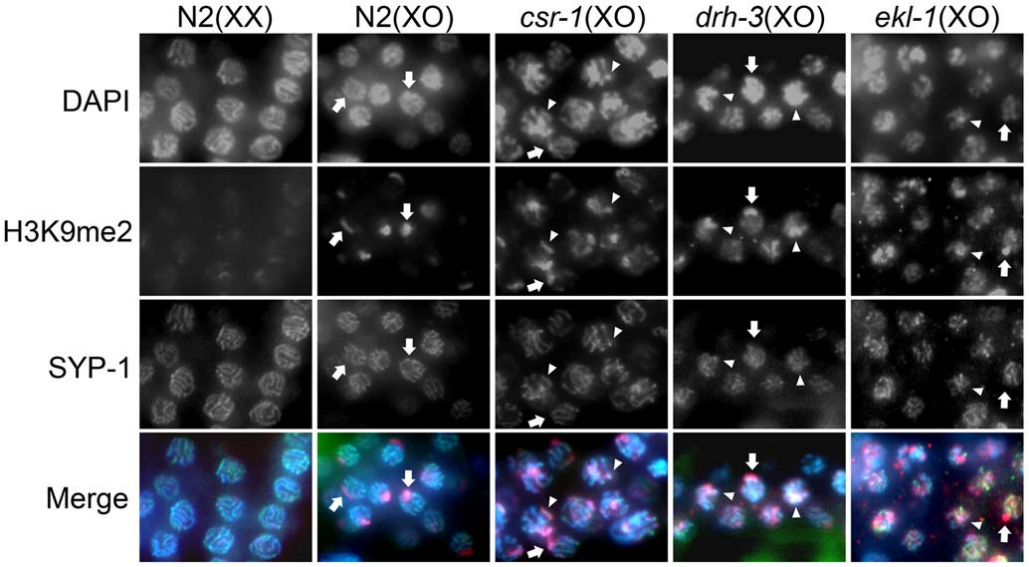
SYP-1 *vs* H3K9me2 distribution in *csr-1*, *ekl-1*, and *drh-3* mutants. Each panel shows pachytene nuclei in an adult germ line co-labeled with DAPI to visualize DNA, anti-SYP-1 to visualize the synaptonemal complex, and anti-H3K9me2. As indicated in Figure S2, Figure S3, and Figure S4, SYP-1 is associated with all chromosomes except the partnerless male X (arrows). In wildtype male germ cells, the strong focus of H3K9me2 staining corresponds to the X chromosome (arrows). In mutants, H3K9me2 foci can be found associated with SYP-1 (arrowheads), indicating H3K9me2 enrichment on synapsed chromosomes. Images were captured on a Zeiss Axioscope.

We also evaluated whether H3K9me2 accumulates at synapsed chromatin in *csr-1*, *ekl-1*, and *drh-3* mutant males. To do so, we co-labeled H3K9me2 and SYP-1 (see Materials and Methods). In wild type males, we consistently observed a single chromosomal region that failed to accumulate SYP-1 and was highly enriched for H3K9me2 (Figure 7). In *csr-1*, *ekl-1*, and *drh-3* mutant males, we typically observed a single SYP-1(-) region that accumulated a variable level of H3K9me2. In addition, we observed H3K9me2 at other chromosomal regions that contained SYP-1 (Figure 7). At the limit of sensitivity of our data, these results are consistent with the hypothesis that elevated H3K9me2 accumulation occurs at synapsed regions in these germ cells.

### EGO-1 is not required for the elevated autosomal H3K9me2 observed in *csr-1*, *ekl-1*, and *drh-3* mutants

Our previous work indicated that the loss of EGO-1 activity prevents H3K9me2 accumulation on unpaired chromatin [9]. Here, we tested whether EGO-1 activity is required for ectopic H3K9me2 accumulation by determining the H3K9me2 distribution in *ego-1;csr-1*, *ego-1 drh-3*, and *ekl-1 ego-1* double mutant males. The H3K9me2 distribution in all three double mutants resembled the distribution we had observed in *csr-1*, *ekl-1*, and *drh-3* single mutant males (Figure 2F, 2G, 2H). Therefore, EGO-1 activity is not necessary for ectopic H3K9me2 to accumulate on autosomes. Moreover, since EGO-1 is required for the H3K9me2 accumulation diagnostic of meiotic silencing, this result strengthens our conclusion that the autosomal H3K9me2 in *csr-1*, *ekl-1*, and *drh-3* males is mis-targeted to paired chromatin. We also note that, in double mutants such as *ekl-1 drh-3*, the H3K9me2 distribution resembled that observed in the two corresponding single mutants (Figure 2I, and data not shown).

### Loss of HIM-17 function combined with CSR-1, EKL-1, or DRH-3 function produces severely abnormal germ lines

We sought to determine whether the pattern of elevated autosomal H3K9me2 in *csr-1*, *ekl-1*, and *drh-3* mutants depended on HIM-17 activity. HIM-17 is a chromatin-associated protein reported to be required for normal accumulation of H3K9me2 *per se* in both XX and XO germ lines [69]. We constructed *him-17;csr-1*, *ekl-1;him-17*, and *drh-3;him-17* double mutants and found that they have severe germline defects similar to those previously described for *ego-1;him-17* double mutants [9] (data not shown). Unfortunately, nuclear morphology was abnormal throughout the severely impaired germ line, prohibiting meaningful interpretation of the H3K9me2 labeling pattern.

## Discussion

Here, we demonstrate that CSR-1, EKL-1, and DRH-3 activities promote the normal accumulation of a chromatin silencing modification, H3K9me2, during meiosis. Our data suggest that, in *csr-1*, *ekl-1*, and *drh-3* mutants, H3K9me2 fails to accumulate to normal levels on chromatin that is unpaired and unsynapsed (e.g., the single male X and the two *him-8* hermaphrodite X chromosomes) and accumulates inappropriately on chromatin that is both paired and synapsed (e.g., autosomes). We interpret these findings to mean that the normal targeting mechanism is disrupted in *csr-1*, *ekl-1*, and *drh-3* mutants. Therefore, CSR-1, EKL-1, and DRH-3 act, directly or indirectly, to target H3K9me2 to appropriate (unpaired) sites and/or prevent accumulation at inappropriate (paired) sites.

### Alternative models for the regulation of H3K9me2 accumulation

Biochemical analysis of CSR-1 and DRH-3 has provided direct insight into their functions. AGO proteins are known to localize to target RNAs via interaction with a siRNA “guide” molecule [38]. Using *in vitro* assays, Aoki *et al*. [46] demonstrated that CSR-1 has Slicer endonuclease activity and binds to secondary (2°) siRNAs that are produced as a consequence of RdRP activity on target mRNA during the RNAi process. DRH-3 activity promotes the formation of diverse classes of small RNAs [34],[35]. *In vitro*, DRH-3 interacts physically with the somatic RdRP, RRF-1, and is required for 2° siRNA production [46]. By analogy, we hypothesize that DRH-3 may promote EGO-1 activity in the germ line.

Although little is known about the biochemical function of EKL-1, we hypothesize that it may bind methylated proteins via its Tudor domains [70]. Tudor domains from several mammalian proteins have been shown to bind methylated peptides *in vitro*, specifically peptides corresponding to histone H3 tails methylated at either lysine 4 or 9 and histone H4 tail methylated at lysine 20 [71]. Similarly, the DNA repair function of *Saccharomyces cerevisiae* RAD9 apparently requires binding to methylated H3 lysine 79 via its Tudor domain [72].

We consider two general models for how an EGO-1/CSR-1/EKL-1/DRH-3 pathway might function in meiotic chromatin regulation. One model is that these factors directly target the chromatin-modifying machinery to unpaired regions, perhaps via a mechanism similar to that which directs H3K9me2 to centromeric repeats in *S. pombe*. There is increasing evidence that siRNAs and other small RNAs participate in transcriptional silencing in many organisms, although thus far the mechanisms are poorly understood [19]. Ultimately, this pathway may establish a self-amplifying loop to attract histone methyltransferase (HMTase) to unpaired chromatin and/or exclude HMTase activity from paired chromatin. We speculate that chromatin-associated RNA pol II transcripts [73]–[76] act as templates for EGO-1 RdRP activity and are essential for establishment of the self-amplifying loop. One possible scenario is that all or a subset of these proteins are initially recruited to unpaired chromatin via interaction with a factor that is lost or masked by successful pairing and/or synapsis. As an amplification loop is established, the HMTase is preferentially recruited to unpaired regions. In the absence of EGO-1 activity, the HMTase may be recruited to specific sites but be unable to methylate chromatin effectively. In the absence of CSR-1, EKL-1, or DRH-3 activity, the HMTase may not be properly recruited or retained and therefore be free to modify chromatin in an unregulated manner, perhaps through enhanced interaction with another competing complex.

As an alternative model, EGO-1, CSR-1, EKL-1, and DRH-3 may influence H3K9me2 by participating in post-transcriptional and/or transcriptional silencing mechanisms that ultimately regulate the expression of genes whose products discriminate between paired and unpaired chromatin. This model is complicated by the fact that we would expect direct targets of such a hypothetical silencing mechanism to be up-regulated upon loss of silencing activity. Therefore, we propose that loss of silencing activity would indirectly down-regulate the discriminatory factors, perhaps by allowing over-expression of a negative regulator. EGO-1 might regulate a different constellation of genes than do CSR-1, EKL-1, and DRH-3, resulting in the different H3K9me2 patterns in *ego-1 versus csr-1*, *drh-3*, and *ekl-1* mutants. Identification of specific sites on unpaired chromatin that are targeted for H3K9me2 accumulation, and investigation of whether EGO-1, CSR-1, EKL-1, and/or DRH-3 associate with those sites will help to distinguish between these alternative models.

### A larger regulatory framework for EGO-1 activity

Our phenotypic analysis of CSR-1, EKL-1, and DRH-3 suggests that they participate in a complex regulatory network to promote development of the germ line. Their activity is critical for maintenance of germline proliferation, meiotic progression, spermatogenesis, and oogenesis. Previous reports in the literature have indicated that EGO-1, EKL-1, DRH-3, and CSR-1 may promote other aspects of development, including embryonic viability and proper chromosome segregation [34],[42],[58]. Most strikingly, Rocheleau *et al*. [63] demonstrated that reduction in function of each of these four genes enhanced the lethality of a weak *ksr-1* (kinase suppressor of ras) mutation. *ksr-1* lethality results from a defect in excretory duct formation due to impaired Ras signaling [77]. Rocheleau *et al.* proposed that EGO-1, CSR-1, DRH-3, and EKL-1 may affect the development of the excretory duct cell by promoting the biogenesis/activity of a set of germline small RNAs whose activity ultimately regulates expression of factors important for the KSR-1/KSR-2 Ras-ERK signaling pathway. We have now demonstrated the importance of this non-coding RNA pathway in meiotic chromatin regulation and other aspects of germline development.

Our genetic data suggest that EGO-1, CSR-1, EKL-1, and DRH-3 participate in a complex regulatory network. Based on strict epistasis criteria, EGO-1 and CSR-1 act in a common genetic pathway to promote bivalent stability at diakinesis, and this pathway works in parallel with DRH-3 and EKL-1 pathways. Given what is known about the biochemical functions of these proteins, perhaps the simplest way to think about these genetic pathways is that they may involve distinct classes of small RNAs (e.g., [35],[45],[78]). The EGO-1/CSR-1/EKL-1/DRH-3 pathway may be responsible for biogenesis/function of one class of small RNA, while other classes of small RNA may require EKl-1 and/or DRH-3, but rely on a different RdRP and/or AGO protein in place of EGO-1 and CSR-1. Indeed, DRH-3 is required for production of many classes of small RNAs, while individual RdRPs function in biogenesis of a subset of such RNAs [34],[35]. Analysis of sperm nuclear morphological detects also suggested a complex pattern of regulation by multiple small RNA-mediated pathways. In this case, the most important pathway(s) require(s) DRH-3 and EKL-1 activity while EGO-1 and CSR-1 activity appear to play only a minor role in this process. Hence, analysis of mutant phenotypes can provide insight into the relationships among different small RNA-mediated pathways.

## Materials and Methods

### Nematode strains and culture

*C. elegans* strains were cultured using standard methods as described [79]. *C. elegans* var Bristol (N2) is the wild type parent strain of all the mutants used in this study. The following mutations, chromosomal deficiencies, duplications, and reciprocal translocations were used: LG (linkage group) I: C04F12.1 *tm1637*, *drh-3(tm1217)*, *drh-3(om55)* (this report), *ego-1(om84)*, *ekl-1(om56, om83)* (this report), F55A12.1 *ok1078*, R06C7.1 *ok1074*, *ppw-1(pk1425)*, *ppw-2(tm1120)*, *prg-1(tm872)*, *sago-2(tm894)*, *sin-3(tm1276)*, T23D8.7 *tm1163, unc-13(e51)*, *unc-15(e73)*, *unc-55(e402)*, *ozDf5*, *nDf25*; LG II: *alg-2(ok304)*, C06A1.4 *tm887*, F58G1.1 *tm1019*, Y49F6A.1 *tm1127*, ZK1248.7 *tm1135*; LG III: C16C10.3 *tm1200*, *tag-76(ok1041)*, *unc-32(e189)*, *zfp-1(ok554)*, *sDf121*, *sDp3 (III;f)*, *hT2[bli-4(e937) let-?(q782) qIs48](I,III)*; LG IV: *csr-1(tm892)*, *drh-1(tm1329)*, *drh-2(tm728)*, *gfl-1(gk321)*, *him-8(e1489)*, *him-17(e2806, ok424)*, M03D4.6 *tm1144*, *prg-2(tm1094)*, T22B3.2 *tm1155*, *nT1[qIs51]*(IV,V); LG V: *ergo-1(tm1860)*, *sago-1(tm1195)*, T22H9.3 *tm1186*, ZK218.8 *tm1324*; LG X: R04A9.2 *tm1116*. The *tm*, *ok*, and *gk* alleles are deletions and therefore likely to be null or extreme loss-of-function. *ego-1(om84)* is a protein null [54]. *ekl-1(om83)* is a deletion allele (this report). An integrated transgenic array, *ccIs4251[myo-3::Ngfp-lacZ*+*myo-3::Mtgfp]*, was used as an LGI marker. Information on specific genes and alleles can be found at Wormbase (http://www.wormbase.org) unless otherwise noted.

Multiple mutant strains were generated using standard genetic strategies. PCR analysis was routinely used to confirm the presence of deletion mutations. The following strategy was used to build *cis-*doubles. To generate *ego-1(om84) drh-3(tm1217)* double mutants, we generated an *ego-1(om84) unc-55(e402)/unc-13(e51) drh-3(tm1217)* male/hermaphrodite strain and mated non-Unc males with *unc-13(e51) unc-55(e402)* hermaphrodites. Non-Unc-13, non-Unc-55 progeny were recovered; PCR analysis was used to identify the lines carrying both *ego-1(om84)* and *drh-3(tm1217)* deletions (i.e., *ego-1 drh-3/unc13 unc-55*). The *ego-1(om84) drh-3(tm1217)* chromosome was then balanced with *hT2[bli-4(e937) let-?(q782) qIs48]*. *ekl-1(om83) ego-1(om84)* and *ekl-1(om83) drh-3(tm1217)* double mutants were constructed by the same general strategy (using different marker mutations in one case).

### RNAi

RNAi was done by the feeding method as described [80] except that double strand RNA production was sometimes induced by 0.2% lactose rather than 1 mM IPTG. Multiple L4 N2 and *glp-1*(*bn18*) hermaphrodites were placed onto each bacterial “feeding” strain at 25°C and 20°C, respectively. Adult F1 progeny were scored for sterility using a dissecting microscope. Steriles were examined at high magnification as described [81] to determine whether they had a Glp-1 sterile phenotype (premature meiotic entry of all germ cells).

### Single nucleotide polymorphism mapping and DNA sequencing

*ego* mutations *om55, om56,* and *om83* were recovered in genetic screens for enhancers of *glp-1(bn18ts)* as previously described [36] using either ethylmethane sulfonate (EMS) (*om55, om56*) or trimethylpsoralen/UV irradiation (*om83*) as the mutagen. Three-factor and deletion mapping placed the three mutations on the right arm of LGI. Based on complementation tests, *om56* and *om83* comprise a single complementation group while *om55* comprises another.

Three-factor mapping placed *om56* and *om83* between *dpy-5* and *unc-13*. We subsequently mapped *om56* relative to single nucleotide polymorphisms (SNPs), ultimately localizing it between SNPs at nucleotide position ∼7050 K and 7120 K. This interval was predicted to encode 19 genes, including *ekl-1* (see www.wormbase.org). DNA from the *ekl-1* gene region was amplified from *ego(om83)* and *ego(om56)* mutants and sequenced. In *ego(om83)* animals, the *ekl-1* open reading frame (ORF) contained a 110 nucleotide deletion and concomitant single nucleotide insertion (at the deletion site); the net 109 nucleotide deletion is predicted to shift the ORF, resulting in production of a truncated product comprising 314 amino acids (Figure 1A). In *ego(om56)* mutants, the *ekl-1* ORF contained a single nucleotide substitution, inserting a stop codon for tryptophan 319 (Figure 1A). Primers used to sequence the *ekl-1* region were (5′→3′): ekl1-1r cgattgcgcgacgaatctgatc; ekl1-2f ggaagttgttctctccactg; ekl1-2r ccgaataagcagtaaactaagg; ekll-3f cactggagagtggcaaagag; ekl1-3r ctccgcacacttgcattgc; ekl1-0r cctgaatagcttgccacgg; ekl1-4f cgttcatttccaacagattg.

Three-factor mapping placed *om55* within an ∼244 kb region between *gld-1* and *unc-55* that includes *drh-3*. *om55* failed to complement *drh-3(tm1217)* for fertility. DNA from the *drh-3* region was amplified from *om55* animals and sequenced using standard methods. A single substitution was detected in the *drh-3* open reading frame (ORF); this change is predicted to replace glycine with glutamic acid at residue 133, leading to production of truncated product (Figure 1B). We conclude that *om55* is an allele of *drh-3*. Primers used to sequence the *drh-3* region were: OM5501F gcattgagatcgaaaggcag; OM5501R catgttgttcaaactggcgc; drh3s1.1f cagagaagattctcggaatg; drh3s1.1r catcacttcgtcagcaattc; drh3s1.2f ggtcgaagatttgctaaccg; drh3s1.2r cggttagcaaatcttcgacc; drh3s2.1f cgaacatcccaaggaaagcc; drh3s2.1r ccaacatgctcattgagctc; drh3s2.2f cgcattgatcaacgctccac; drh3s2.2r caagcatagttcgacagctg; drh3s2.3f ggtctgacagcttcattgag; drh3s3.1f ggtctcgatgttactgcatg; drh3s3.1r gcggcaaataggttcctctg; drh3s3.2f catggtgttcgatccaagtg; drh-3s3.2r gatcgaatgaaaattgctcgg.

### Indirect immunofluorescence

H3K9me2 single labeling was carried out as described [9] using polyclonal anti-H3K9me2 (gift of C. D. Allis) at 1/500 dilution and Alexa488-labeled secondary antibody (Invitrogen) at 1∶200 dilution. H3K9me2/SYP-1 double labeling was performed as follows. Gonads were dissected in 8 µL of 0.25 mM levamisole/PBS on a poly-lysine treated slide. 8 µL of 6% paraformaldehyde (PFA)/2X EGG buffer were added to the dissected tissue and a Super-Frost slide (Fisher) immediately placed on top. The slide sandwich was placed on dry ice for 15 minutes, cracked open, and immediately washed with PBST. After a total of 3X 5 min washes in PBST (1X PBS/0.1% Tween-20), the sample was blocked for 30 minutes in 30% goat serum (GS)/PBST. Monoclonal anti-H3K9me2 (1∶200 dilution, Abcam1220) and polyclonal anti-SYP-1 (1∶200 dilution, STD143 gift of A. Villeneuve) were added. Tissue was incubated at 4°C overnight and then washed 3X 10 min in PBST. Tissue was incubated with Alexa488-conjugated goat anti-rabbit (1∶200 dilution, Invitrogen) and Alexa568-conjugated goat anti-mouse (1∶400 dilution, Invitrogen) secondary antibodies for 2 hours at room temperature and then washed 1X in PBST, 2X in PBS. DAPI was added to the first PBS wash. Images were captured on a Zeiss Axioscope and, in some cases, on a Zeiss LSM 710 Confocal microscope.

H3K4me2 and H3K9me2 co-labeling was performed using rabbit anti-H3K4me (gift of C. D. Allis) and mouse anti-H3K9me2 (Abcam 1220). Dissected tissue was fixed for 5 min in 2.5% PFA, washed 3X in PBST, blocked >30 min in PBST/GS, and incubated overnight at room temp in primary antibody diluted 1∶200 (anti-H3K9me2) or 1∶250 (anti-H3K4me2) in PBST/GS. Washes and secondary antibody staining was carried out as described above. HIM-3 and SYP-1 co-labeling was performed using a similar protocol, except that dissected gonads were fixed for 5 min in 1% PFA and post-fixed for 1 min with −20°C methanol prior to PBST washes. Rabbit anti-HIM-3 (gift of M. Zetka) and guinea pig anti-SYP-1 (STD 165, gift of A. Villeneuve) were each diluted 1/200. Alexa488-conjugated goat anti-guinea pig (Invitrogen) was diluted 1/200.

### Fluorescent *in situ* hybridization

The 5S rDNA probe was generated by amplification of a 1 kb region of the 5S rDNA locus using published primers [82]. The probe was labeled with DIG-11-dUTP using the DIG-Nick Translation Kit (Roche Applied Science). FISH was carried out as described [8]. A 1∶200 dilution of anti-Digoxigenin-Fluorescein antibody (Roche Applied Science) was used for probe detection. Samples were examined using a DRMXA fluorescent microscope (Leica); the images were acquired using a CCD camera (Q Imaging) and processed using SimplePCI (Hamamutsu Corporation) software.

### Phenotypic characterization

DAPI staining was used to characterize the germline developmental phenotypes. To avoid variations in germline morphology caused by aging, animals were harvested at a consistent developmental stage (24 hours post-L4 stage at 20°C). Animals were then dissected to expose the gonad. Fixation and staining were performed as described [83]. Nuclei in mitosis and different stages of meiosis were identified based on nuclear morphology as described [37],[83].

## Supporting Information

Figure S1 Relative distribution of H3K9me2 and H3K4me2 in *him-8* XX germlines. Panels show meiotic nuclei co-labeled with DAPI to visualize DNA and polyclonal antibody against H3K9me2 and H3K4me2. In *him-8* XX germ cells (as in wildtype), H3K4me2 is not detected on the X chromosomes (arrowheads) and is present at a high level on autosomes. In contrast, H3K9me2 levels are high on the unpaired/unsynapsed X chromosomes (arrowheads) and very low on autosomes. In *csr-1*, *ekl-1*, and *drh-3* mutants, one chromosomal region lacks H3K4me2 and contains a variable level of H3K9me2 (arrowheads); this is presumably the X chromosome. Arrow indicates a chromosome that lacks both H3K4me2 and H3K9me2. Other chromosomes are enriched for H3K4me2 and contain a variable (low) level of H3K9me2. Images were captured on a Zeiss Axioscope.
(3.11 MB TIF)

Figure S2 HIM-3 and SYP-1 distribution in *csr-1*, *ekl-1*, and *drh-3* XO mutants. Each panel shows pachytene nuclei from an XO germ line co-labeled with DAPI to visualize DNA and with polyclonal antisera to visualize HIM-3 and SYP-1. HIM-3 associates with all chromosomes. A single region fails to accumulate SYP-1 (arrowheads), which is presumably the X chromosome. The arrow in the *csr-1* image indicates an example of the large abnormal nuclei we also observe in *ekl-1*, *drh-3*, and *ego-1* mutants. See Text S1. Full genotypes were: *him-8, csr-1*, *ekl-1;him-8*, and *drh-3;him-8*. Images were captured on a Zeiss LSM 710 confocal microscope.
(10.28 MB TIF)

Figure S3 Co-localization of HIM-3 and SYP-1 on pachytene chromosomes in XX *csr-1* and *ekl-1* mutants. Each panel shows pachytene nuclei from an XX germ line co-labeled with DAPI (A,E,I) to visualize DNA and with polyclonal antisera to visualize HIM-3 (B,F,J) and SYP-1 (C,G,K). (D,H,L) Merged SYP-1 and HIM-3 images. (A-D,I-L) HIM-3 and SYP-1 labeling is co-linear in N2 wildtype and *ekl-1* nuclei. (E–H) Some *csr-1* nuclei contain chromosomal regions with only HIM-3 or only SYP-1 (arrows). (I–J) *ekl-1* image contains an example of a large, putative “polyploidy” nucleus (arrow). Images were captured on a Zeiss LSM 710 confocal microscope.
(9.20 MB TIF)

Figure S4 Co-localization of HIM-3 and SYP-1 on pachytene chromosomes in XX *drh-3;him-8* mutants. Each panel shows pachytene nuclei from an XX germ line co-labeled with DAPI (A,E) to visualize DNA and with polyclonal antisera to visualize HIM-3 (B,F) and SYP-1 (C,G). (D,H) Merged SYP-1 and HIM-3 images. (A–D) *him-8* and (E–H) *drh-3;him-8* nuclei contain 1–2 regions that lack SYP-1 (arrows), which presumably correspond to the X chromosomes. Images were captured on a Zeiss LSM 710 confocal microscope.
(5.52 MB TIF)

Table S1 Distribution of LGV FISH signals in *csr-1*, *ekl-1*, and *drh-3* mutants. In *csr-1*, *ekl-1*, and *drh-3* mutants, nuclei with abnormal chromosomal morphology are scattered within the pachytene zone as discussed in Text S1. The number of FISH foci was counted in each nucleus within the pachytene zone regardless of chromosomal morphology. Independent values are given for XX and XO germ lines. N, the number of pachytene zone nuclei that were counted.
(0.04 MB DOC)

Table S2 Single LGV FISH signals in morphologically pachytene nuclei. The number of FISH foci was counted in nuclei with recognizable pachytene morphology. Independent values are given for XX and XO germ lines. See Text S1 for discussion. N, number of nuclei counted.
(0.03 MB DOC)

Table S3 The majority of large, diffuse nuclei within the pachytene zone contained multiple FISH foci. The percent of abnormal, large nuclei containing multiple FISH foci is indicated. Independent values are given for XX and XO germ lines. Note that some abnormal nuclei contained only a single FISH signal. See Text S1 for discussion. N, number of nuclei counted. NA, not applicable.
(0.03 MB DOC)

Text S1 Supplemental information and references.
(0.04 MB DOC)
