# Supplementary figures and images for: Regulation of Heterochromatin Assembly on Unpaired Chromosomes during Caenorhabditis elegans Meiosis by Components of a Small RNA-Mediated Pathway
Source: PLoS Genet. 2009 Aug 28;5(8):e1000624. doi: 10.1371/journal.pgen.1000624 (PMC2726613; doi:10.1371/journal.pgen.1000624)

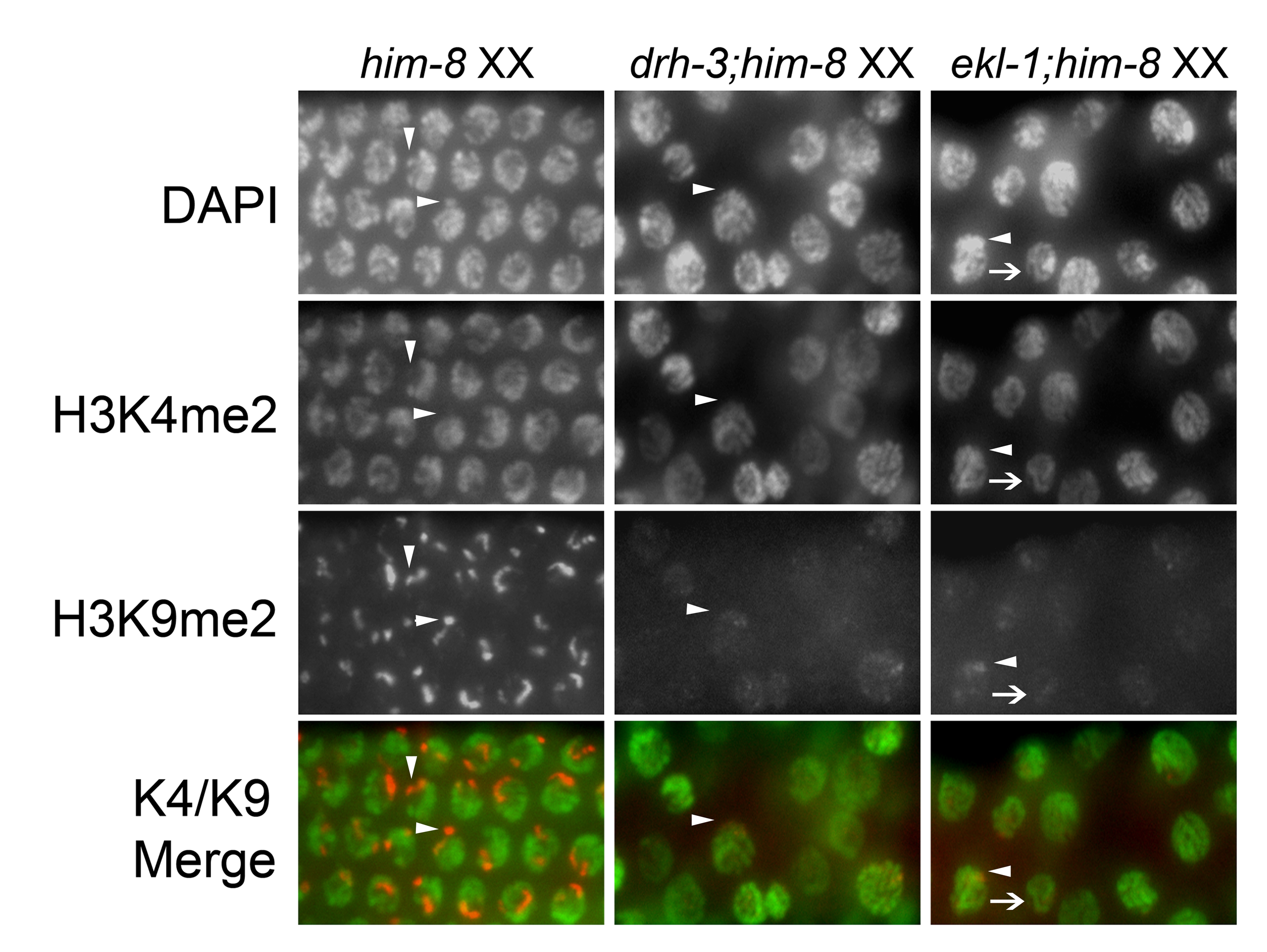

Supplement: Figure S1 — Relative distribution of H3K9me2 and H3K4me2 in him-8 XX germlines. Panels show meiotic nuclei co-labeled with DAPI to visualize DNA and polyclonal antibody against H3K9me2 and H3K4me2. In him-8 XX germ cells (as in wildtype), H3K4me2 is not detected on the X chromosomes (arrowheads) and is present at a high level on autosomes. In contrast, H3K9me2 levels are high on the unpaired/unsynapsed X chromosomes (arrowheads) and very low on autosomes. In csr-1, ekl-1, and drh-3 mutants, one chromosomal region lacks H3K4me2 and contains a variable level of H3K9me2 (arrowheads); this is presumably the X chromosome. Arrow indicates a chromosome that lacks both H3K4me2 and H3K9me2. Other chromosomes are enriched for H3K4me2 and contain a variable (low) level of H3K9me2. Images were captured on a Zeiss Axioscope. (3.11 MB TIF) [file pgen.1000624.s001.tif]

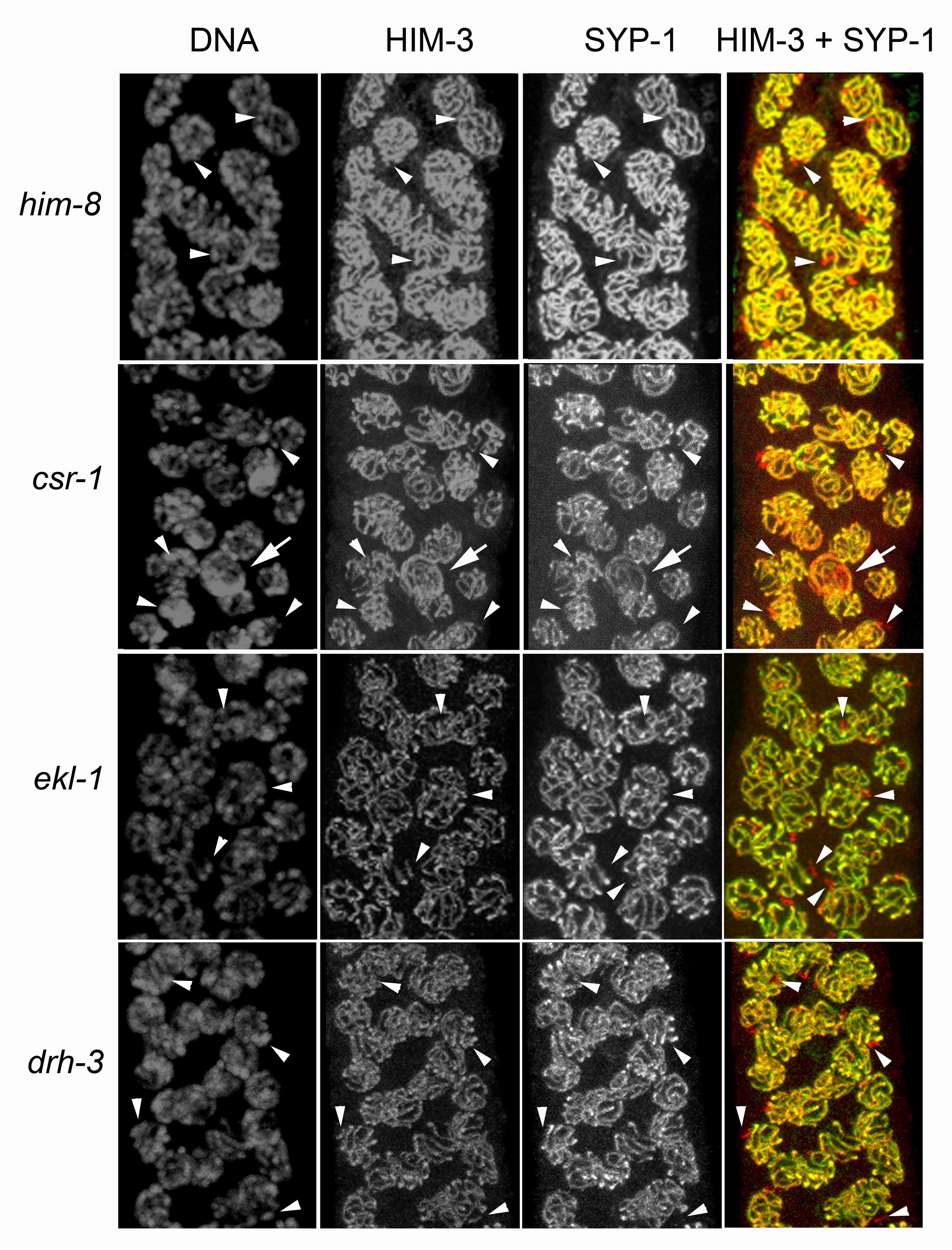

Supplement: Figure S2 — HIM-3 and SYP-1 distribution in csr-1, ekl-1, and drh-3 XO mutants. Each panel shows pachytene nuclei from an XO germ line co-labeled with DAPI to visualize DNA and with polyclonal antisera to visualize HIM-3 and SYP-1. HIM-3 associates with all chromosomes. A single region fails to accumulate SYP-1 (arrowheads), which is presumably the X chromosome. The arrow in the csr-1 image indicates an example of the large abnormal nuclei we also observe in ekl-1, drh-3, and ego-1 mutants. See Text S1. Full genotypes were: him-8, csr-1, ekl-1;him-8, and drh-3;him-8. Images were captured on a Zeiss LSM 710 confocal microscope. (10.28 MB TIF) [file pgen.1000624.s002.tif]

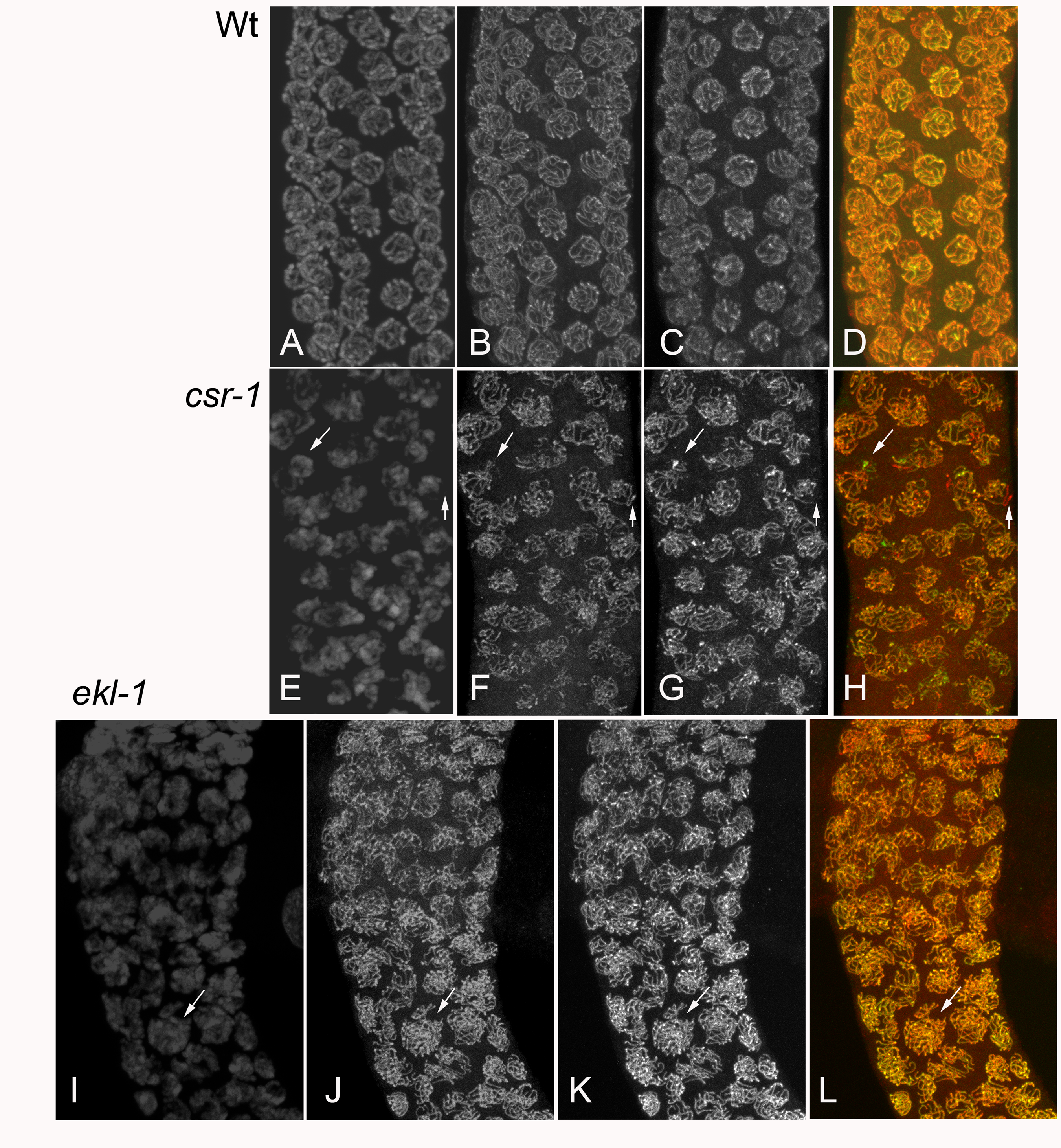

Supplement: Figure S3 — Co-localization of HIM-3 and SYP-1 on pachytene chromosomes in XX csr-1 and ekl-1 mutants. Each panel shows pachytene nuclei from an XX germ line co-labeled with DAPI (A,E,I) to visualize DNA and with polyclonal antisera to visualize HIM-3 (B,F,J) and SYP-1 (C,G,K). (D,H,L) Merged SYP-1 and HIM-3 images. (A-D,I-L) HIM-3 and SYP-1 labeling is co-linear in N2 wildtype and ekl-1 nuclei. (E–H) Some csr-1 nuclei contain chromosomal regions with only HIM-3 or only SYP-1 (arrows). (I–J) ekl-1 image contains an example of a large, putative “polyploidy” nucleus (arrow). Images were captured on a Zeiss LSM 710 confocal microscope. (9.20 MB TIF) [file pgen.1000624.s003.tif]

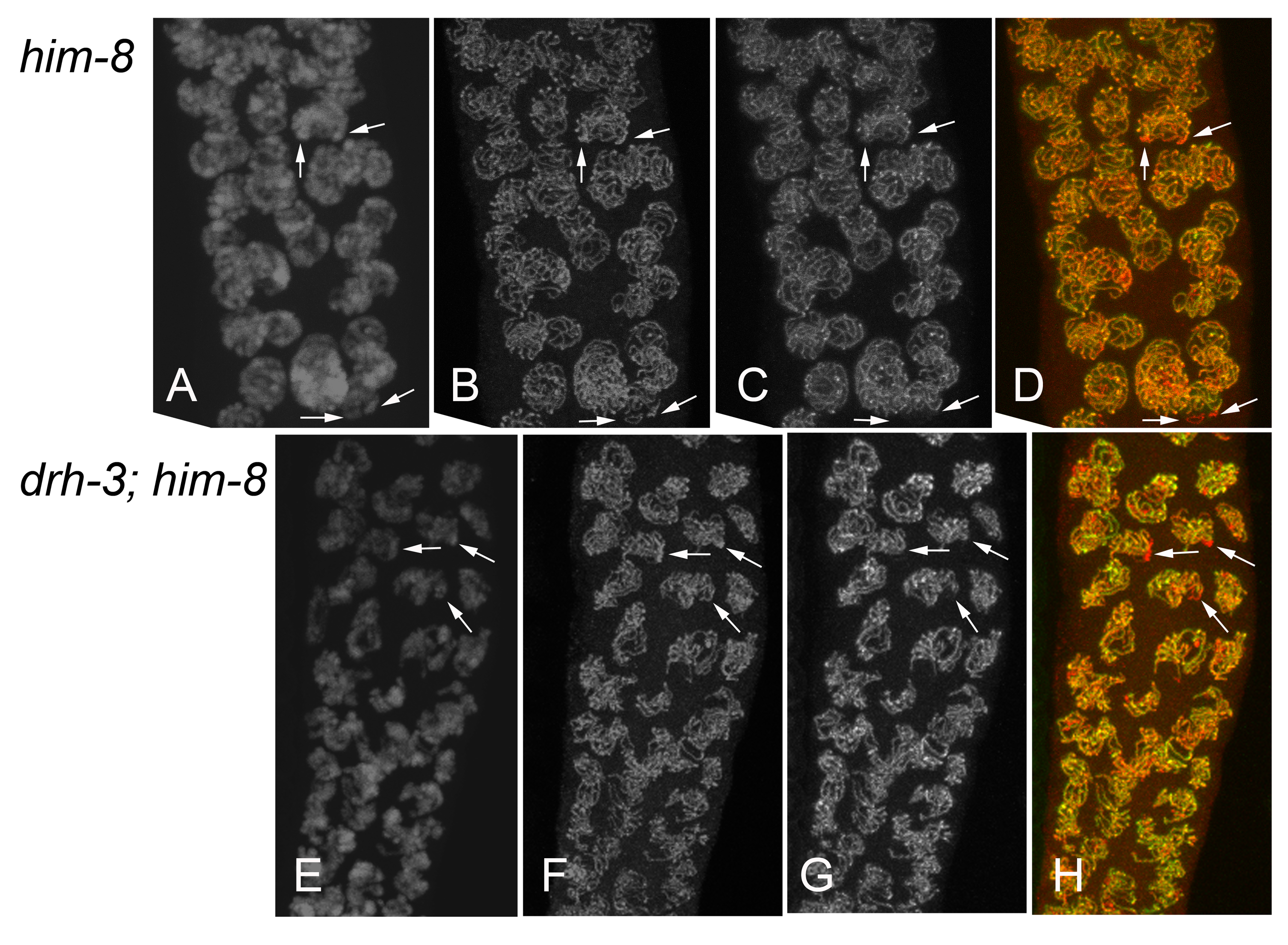

Supplement: Figure S4 — Co-localization of HIM-3 and SYP-1 on pachytene chromosomes in XX drh-3;him-8 mutants. Each panel shows pachytene nuclei from an XX germ line co-labeled with DAPI (A,E) to visualize DNA and with polyclonal antisera to visualize HIM-3 (B,F) and SYP-1 (C,G). (D,H) Merged SYP-1 and HIM-3 images. (A–D) him-8 and (E–H) drh-3;him-8 nuclei contain 1–2 regions that lack SYP-1 (arrows), which presumably correspond to the X chromosomes. Images were captured on a Zeiss LSM 710 confocal microscope. (5.52 MB TIF) [file pgen.1000624.s004.tif]
