# Supplementary material for: Regulation of Heterochromatin Assembly on Unpaired Chromosomes during Caenorhabditis elegans Meiosis by Components of a Small RNA-Mediated Pathway
Source: PLoS Genet. 2009 Aug 28;5(8):e1000624. doi: 10.1371/journal.pgen.1000624 (PMC2726613; doi:10.1371/journal.pgen.1000624)
Supplement: Table S1 — Distribution of LGV FISH signals in csr-1, ekl-1, and drh-3 mutants. In csr-1, ekl-1, and drh-3 mutants, nuclei with abnormal chromosomal morphology are scattered within the pachytene zone as discussed in Text S1. The number of FISH foci was counted in each nucleus within the pachytene zone regardless of chromosomal morphology. Independent values are given for XX and XO germ lines. N, the number of pachytene zone nuclei that were counted. (0.04 MB DOC) [file pgen.1000624.s005.doc]

**Table S1.** Distribution of LGV FISH signals in *csr-1*, *ekl-1*, and *drh-3* mutants.

| **Genotype** | **Gender** | **% 1 signal** | **% 2 signal** | **% >2 signal** | **N** |
| --- | --- | --- | --- | --- | --- |
| **Wildtype** | XX | 95 | 5 | 0 | (61) |
|  | XO | 100 | 0 | 0 | (80) |
| ***csr-1*** | XX | 96 | 4 | 0 | (50) |
|  | XO | 76 | 19 | 5 | (38) |
| ***ekl-1*** | XX | 81 | 10 | 9 | (113) |
|  | XO | 91 | 7 | 2 | (43) |
| ***drh-3*** | XX | 62 | 38 | 0 | (45) |
|  | XO | 72 | 23 | 5 | (56) |
