# Supplementary material for: Regulation of Heterochromatin Assembly on Unpaired Chromosomes during Caenorhabditis elegans Meiosis by Components of a Small RNA-Mediated Pathway
Source: PLoS Genet. 2009 Aug 28;5(8):e1000624. doi: 10.1371/journal.pgen.1000624 (PMC2726613; doi:10.1371/journal.pgen.1000624)
Supplement: Table S2 — Single LGV FISH signals in morphologically pachytene nuclei. The number of FISH foci was counted in nuclei with recognizable pachytene morphology. Independent values are given for XX and XO germ lines. See Text S1 for discussion. N, number of nuclei counted. (0.03 MB DOC) [file pgen.1000624.s006.doc]

**Table S2.** Single LGV FISH signals in morphologically pachytene nuclei.

| **Genotype** | **Gender** | **% 1 signal** | **N** |
| --- | --- | --- | --- |
| **Wildtype** | XX | 95 | (61) |
|  | XO | 100 | (80) |
| ***csr-1*** | XX | 100 | (37) |
|  | XO | 100 | (19) |
| ***ekl-1*** | XX | 95 | (76) |
|  | XO | 100 | (36) |
| ***drh-3*** | XX | 68 | (28) |
|  | XO | 82 | (38) |
