# Supplementary material for: Regulation of Heterochromatin Assembly on Unpaired Chromosomes during Caenorhabditis elegans Meiosis by Components of a Small RNA-Mediated Pathway
Source: PLoS Genet. 2009 Aug 28;5(8):e1000624. doi: 10.1371/journal.pgen.1000624 (PMC2726613; doi:10.1371/journal.pgen.1000624)
Supplement: Table S3 — The majority of large, diffuse nuclei within the pachytene zone contained multiple FISH foci. The percent of abnormal, large nuclei containing multiple FISH foci is indicated. Independent values are given for XX and XO germ lines. Note that some abnormal nuclei contained only a single FISH signal. See Text S1 for discussion. N, number of nuclei counted. NA, not applicable. (0.03 MB DOC) [file pgen.1000624.s007.doc]

**Table S3.** The majority of morphologically abnormal nuclei within the pachytene zone contained multiple FISH foci.

| **Genotype** | **Gender** | **% > 2 signals** | **N** |
| --- | --- | --- | --- |
| **Wildtype** | XX | NA | - |
|  | XO | NA | - |
| ***csr-1*** | XX | NA | - |
|  | XO | 100 | (3) |
| ***ekl-1*** | XX | 77 | (22) |
|  | XO | 57 | (7) |
| ***drh-3*** | XX | 100 | (3) |
|  | XO | 80 | (10) |
