# Supplementary material for: Regulation of Heterochromatin Assembly on Unpaired Chromosomes during Caenorhabditis elegans Meiosis by Components of a Small RNA-Mediated Pathway
Source: PLoS Genet. 2009 Aug 28;5(8):e1000624. doi: 10.1371/journal.pgen.1000624 (PMC2726613; doi:10.1371/journal.pgen.1000624)
Supplement: Text S1 — Supplemental information and references. (0.04 MB DOC) [file pgen.1000624.s008.doc]

**Text S1**

Homolog pairing in *csr-1*, *ekl-1*, and *drh-3* mutants

To assay pairing, we performed FISH using a 5S rRNA gene probe to detect LGV (see Materials and Methods). In wild type germ lines, we detected one FISH signal per nucleus in 100% of XO and 95% of XX nuclei as expected when the two LGV homologs are paired (Table S1). We also detected one FISH signal in the majority of *csr-1*, *ekl-1*, and *drh-3* mutant nuclei, although the percent of nuclei with two FISH signals was higher than in wild type in all cases except *csr-1* hermaphrodites (Table S1). Theoretically, the presence of two FISH signals should indicate that the two LGV homologs have failed to pair. In males, we observed two FISH signals in 7% of *ekl-1*, 19% of *csr-1*, and 23% of *drh-3* nuclei; in hermaphrodites, we observed two FISH signals in 4% of *csr-1*, 10% of *ekl-1*, and 38% of *drh-3* nuclei (Table S1). Hence, these mutants appear to have an incompletely penetrant pairing defect. (We note that these data may under represent the pairing defect, since we only assayed one chromosome.) As described above, HIM-3 and SYP-1 double labeling experiments provided no evidence of chromosomes that failed to synapse in these mutants. Therefore, we hypothesize that the unpaired LGV homologs have heterologously synapsed with another (unpaired) chromosome.

We were particularly interested in the pairing status of chromosomes in nuclei with normal pachytene morphology, as we hypothesize that these nuclei are most likely to provide relevant information about chromatin regulation. We categorized nuclei based on morphology and quantified the prevalence of 1, 2, or >2 FISH signals in each type of nucleus. Most nuclei with recognizable pachytene morphology contained one FISH signal (e.g., 100% of *csr-1*, 100% of *ekl-1*, and 82% of *drh-3* male pachytene nuclei) (Table S2). Therefore, the autosomal H3K9me2 accumulation in these nuclei does not appear to result from a pairing defect. Nuclei with abnormal morphology were less likely to have 1 FISH signal; they constituted the majority of nuclei with 2 and all of the nuclei with >2 FISH signals (Table S3 and data not shown). We noted that large abnormal nuclei were particularly likely to have a relatively high level of H3K9me2 (78%-85%; Table 2) and > 2 FISH signals (57%-100%; Table S3). If these nuclei are polyploid, as we suspect, then the presence of additional chromosomes may account for the additional FISH signals and elevated H3K9me2 levels that we often observe. Importantly, ectopic autosomal H3K9me2 is not restricted to abnormal nuclei but is also observed in nuclei with recognizable pachytene morphology (see Figure 3).

Analysis of synaptonemal complex integrity

To visualize the synaptonemal complex (SC), dissected gonads were labeled for HIM-3 and SYP-1 (see Materials and Methods). HIM-3 is an axial component that associates with all chromosomes early in first meiotic prophase prior to SC formation [S1]. SYP-1 is a central element of the SC that associates with chromosomes after HIM-3 has loaded; it does not associate with the male X [S2]. Later, SYP-1 dissociates from chromosomes in an asymmetric fashion and prior to HIM-3 [S3].

In control males, meiotic nuclei contained a single region of HIM-3(+)/ SYP-1(-) chromatin that presumably corresponded to the X chromosome(s) (Figure S2). In *ekl-1*, *drh-3*, and *csr-1* male pachytene nuclei, a single HIM-3(+)/ SYP-1(-) region also was visible (Figure S2). HIM-3 and SYP-1 appeared to co-localize on all other chromosomes. We did not observe large SYP-1(-) regions that would indicate additional chromosomes that had failed to pair and synapse, nor did we observe gaps in the SYP-1 localization pattern that might indicate defects in SC assembly. We cannot, however, rule out subtle defects in SC formation. We also note that analysis of *csr-1* mutants was complicated by the fact that these germ lines were generally smaller and more disorganized in appearance than *drh-3* and *ekl-1* germ lines. All *csr-1* germ lines contained pachytene nuclei that appeared normal. In many *csr-1* germ lines, a distinct pachytene zone was visible (as shown in Figure S2), and we draw our conclusions about SC assembly based on analysis of these germ lines. For a subset of *csr-1* germ lines, we observed that the region with fully loaded SYP-1 was very short and/or pachytene and diplotene nuclei appeared to be interspersed, making it difficult to distinguish between (i) the natural loss of SYP-1 from chromosomes as they progress through late pachytene/diplotene and (ii) chromosomes that did not load SYP-1 in the first place.

In wild type hermaphrodite controls, all pachytene chromosomes were positive for both HIM-3 and SYP-1 (Figure S3A-D). Similarly, HIM-3 and SYP-1 co-localized on pachytene chromosomes in *ekl-1* and *drh-3* mutant hermaphrodites (Figure S3I-L and data not shown). Analysis of *csr-1* in hermaphrodites, as in males, was complicated by the disorganization of the germ line. In all *csr-1* XX germ lines, we observed pachytene nuclei with co-linear labeling of HIM-3 and SYP-1 (Figure S3E-H). In addition, the pachytene zone often contained some nuclei where one or more chromosomal regions lacked SYP-1 (Figure S3F-H); these regions were variably sized compared with the unpaired Xs observed in *him-8* hermaphrodites (Figure S4B-D). Often, they appeared too small to correspond to a pair of chromosomes. These regions may have failed to synapse properly or, alternatively, may have lost SYP-1 as a consequence of the normal chromosome reorganization normally observed in diplotene (perhaps occurring prematurely in *csr-1*?). If these nuclei do in fact contain chromatin that is both unpaired and unsynapsed, then it would normally be targeted for H3K9me2 accumulation by the meiotic silencing machinery; we point out such accumulation was not observed.

We sought to confirm our XX observations in *him-8* hermaphrodites. In *him-8* controls, the unpaired X chromosomes appeared as either one or two distinct SYP-1(-) regions depending on their relative positions in the nucleus (Figure S4A-D). In *drh-3;him-8* and *ekl-1;him-8* hermaphrodites, we observed one or two SYP-1(-) regions of chromatin per nucleus (Figure S4E-H and data not shown). We did not observe additional unpaired chromosomes or gaps in the SYP-1 localization pattern that might indicate defects in SC assembly.

**Supplemental References**

S1. Zetka M Kawasaki I, Strome S, Müller F (1999) Synapsis and chiasma formation in Caenorhabditis elegans require HIM-3, a meiotic chromosome core component that functions in chromosome segregation. Genes Dev. 13:2258-2270.

S2. MacQueen AJ, Colaiacovo MP, McDonald K, Villeneuve AM (2002) Synapsis-dependent and -independent mechanisms stabilize homolog pairing during meiotic prophase in *C. elegans*. Genes Dev 16: 2428-2442.

S3. Nabeshima K, Villeneuve AM, Colaiacovo MP (2005) Crossing over is coupled to late meiotic prophase bivalent differentiation through asymmetric disassembly of the SC. J Cell Biol 168:683-689.
